# Influence of Transmembrane Helix Mutations on Cytochrome P450-Membrane Interactions and Function

**DOI:** 10.1016/j.bpj.2018.12.014

**Published:** 2019-01-03

**Authors:** Ghulam Mustafa, Prajwal P. Nandekar, Tyler J. Camp, Neil J. Bruce, Michael C. Gregory, Stephen G. Sligar, Rebecca C. Wade

**Affiliations:** 1 Molecular and Cellular Modeling Group, Heidelberg Institute for Theoretical Studies, Heidelberg, Germany; 2 Zentrum für Molekulare Biologie der Universität Heidelberg, DKFZ-ZMBH Alliance, Heidelberg, Germany; 3 Department of Biochemistry, University of Illinois at Urbana-Champaign, Urbana, Illinois; 4 Department of Chemistry, University of Illinois, Urbana, Illinois; 5 Interdisciplinary Center for Scientific Computing, Heidelberg University, Heidelberg, Germany

## Abstract

Human cytochrome P450 (CYP) enzymes play an important role in the metabolism of drugs, steroids, fatty acids, and xenobiotics. Microsomal CYPs are anchored in the endoplasmic reticulum membrane by an N-terminal transmembrane (TM) helix that is connected to the globular catalytic domain by a flexible linker sequence. However, the structural and functional importance of the TM-helix is unclear because it has been shown that CYPs can still associate with the membrane and have enzymatic activity in reconstituted systems after truncation or modification of the N-terminal sequence. Here, we investigated the effect of mutations in the N-terminal TM-helix residues of two human steroidogenic enzymes, CYP 17A1 and CYP 19A1, that are major drug targets for cancer therapy. These mutations were originally introduced to increase the expression of the proteins in *Escherichia coli.* To investigate the effect of the mutations on protein-membrane interactions and function, we carried out coarse-grained and all-atom molecular dynamics simulations of the CYPs in a phospholipid bilayer. We confirmed the orientations of the globular domain in the membrane observed in the simulations by linear dichroism measurements in a Nanodisc. Whereas the behavior of CYP 19A1 was rather insensitive to truncation of the TM-helix, mutations in the TM-helix of CYP 17A1, especially W2A and E3L, led to a gradual drifting of the TM-helix out of the hydrophobic core of the membrane. This instability of the TM-helix could affect interactions with the allosteric redox partner, cytochrome b5, required for CYP 17A1’s lyase activity. Furthermore, the simulations showed that the mutant TM-helix influenced the membrane interactions of the CYP 17A1 globular domain. In some simulations, the mutated TM-helix obstructed the substrate access tunnel from the membrane to the CYP active site, indicating a possible effect on enzyme function.

## Introduction

Cytochrome P450s (CYPs) are heme proteins that are ubiquitously present in all kingdoms of life. Mammalian CYPs play an important role in steroidogenesis and in the metabolism of drugs and xenobiotics. Here, we study CYP 17A1 (steroid 17-*α*-hydroxylase/17,20 lyase, EC:1.14.14.19, Uniprot P05093) and CYP 19A1 (aromatase, EC:1.14.14.14, Uniprot P11511), steroidogenic enzymes located in the endoplasmic reticulum (ER) of the adrenal cortex, testes, and ovaries (1). CYP 17A1 has two functions: acting as a 17*α*-hydroxylase in the zona fasciculata of the adrenal gland and as a 17*α*-hydroxylase and 17,20-lyase in the gonads and the adrenal zona reticularis. The lyase reaction results in the conversion of pregnenolone to dehydroepiandrosterone, which leads to the formation of androgens (2). CYP 19A1 catalyzes the formation of estrogen from androgens through a multistep process involving several hydroxylations. The hydroxylase reaction, which is catalyzed by hepatic CYPs as well as steroidogenic CYPs, requires the electron donor, NADPH cytochrome P450 oxidoreductase (CPR), whereas cytochrome b5 (Cyt-b5), which is colocalized with CYP 17A1 in the adrenal zona reticularis and gonads, is involved in the lyase reaction (3).

**Figure 1:**
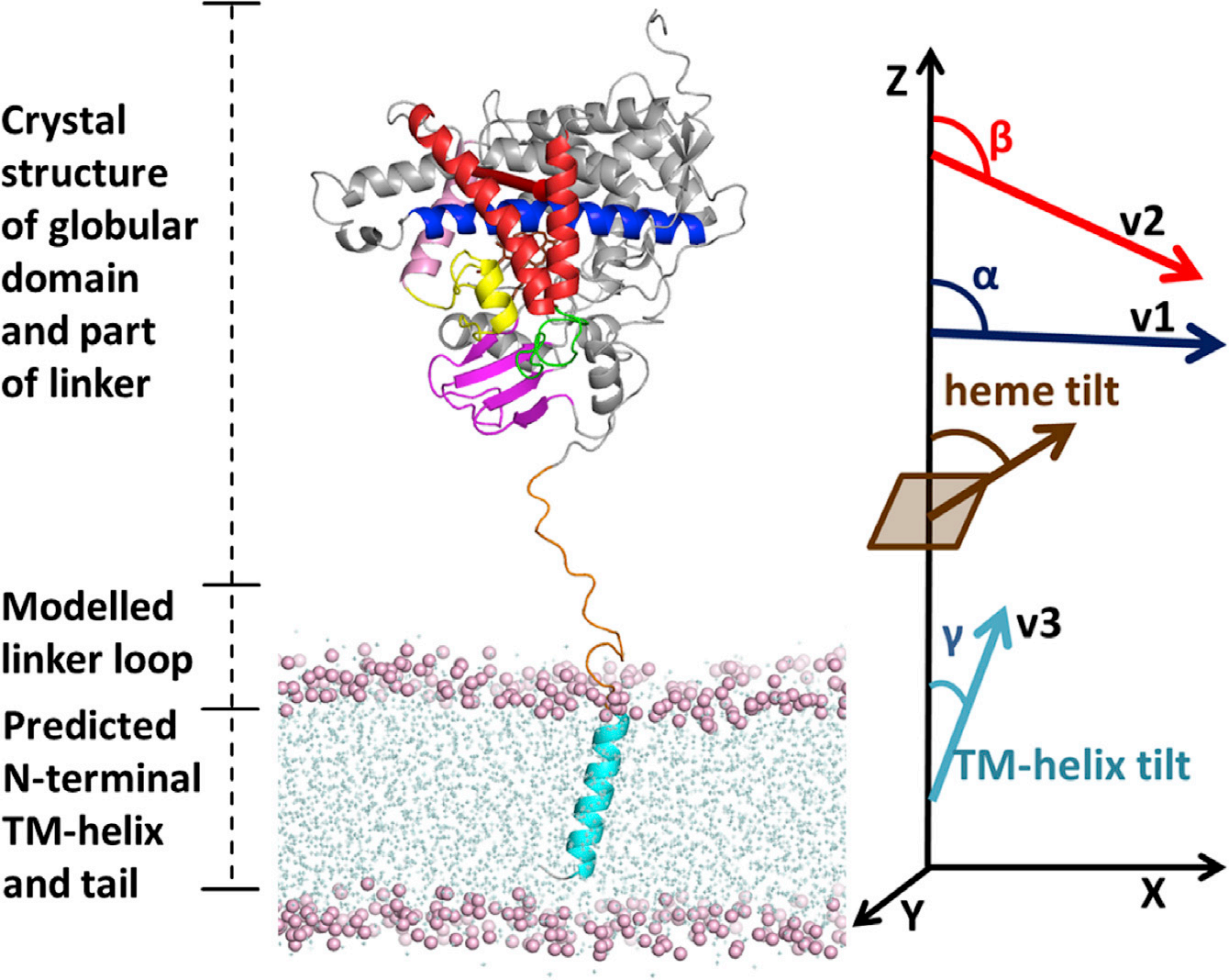
Initial model of wild-type CYP 17A1 (wtCYP17) showing its three domains and the initial information on which modeling and simulation of its arrangement in the bilayer was based. The crystal structure (PDB: 3RUK, chain C) of the globular domain (residues 50–502) and part of the linker (residues 31–49) is shown. Secondary structure predictions indicate the length of the N-terminal TM-helix (*cyan*, residues 3–19). These two components are connected by a modeled linker loop of unknown conformation (*orange*, residues 20–30). The flexible C-terminal tail (residues 503–508) is not included in the model. Important secondary structure elements in the globular domain are colored as follows: *β*-strands 1–4 are in magenta, the F- and G-helices are in red, the I-helix is in blue, the B′-C′ loop is in yellow, and the F-G loop is in green. Experimentally, it is known that the globular domain interacts with the bilayer, and during the CG simulations, it approaches and dips into the bilayer. The angles defining its orientation in the bilayer are shown on the right, along with the definition of the TM-helix tilt angle; the definitions of these angles are given in the Materials and Methods. To see this figure in color, go online.

Microsomal CYPs are anchored in the ER membrane by an N-terminal transmembrane (TM) helix that is connected to the catalytic globular domain by a flexible linker sequence (see Fig. 1). Additionally, hydrophobic patches in the globular domain contribute to its attachment to the ER membrane. In CYPs, the TM-helix generally contains polar, aromatic, or charged residues that flank the hydrophobic residues on both sides of the TM domain. They are important for stabilization of the TM-helix in the bilayer and for protein sorting and retention of CYPs in the ER membrane (4). For example, it has been observed that retention of the TM-helix of CYP 2C1 in the ER membrane is dependent on the hydrophobicity of the TM domain (residues 3–20) and on the hydrophilic residues (21–23 KQS) at the beginning of the linker region (residues 21–28). Mutation of the linker, the hydrophobic TM domain, or both regions resulted in the expulsion of CYP 2C1 from the ER membrane and affected direct retrieval from the pre-Golgi compartment (5). However, apart from the TM-helix, the linker residues and the hydrophobic region in the catalytic domain, particularly the F-G loop, are responsible for the retention of CYPs in the ER membrane (5, 6, 7).

For the purpose of biochemical studies, mammalian CYPs have been mutated at the N-terminus to obtain good expression levels in *Escherichia coli*. For example, Sagara et al. (8) truncated the N-terminal hydrophobic anchor of bovine CYP 17, and Imai et al. (9) introduced N-terminal mutations in CYP 17A1, both obtaining good expression levels in *E. coli* membranes while retaining catalytic activity. Further mutations have been introduced to obtain soluble forms of mammalian CYPs for crystallographic studies. Usually, these involve removing hydrophobic N-terminal residues and hydrophobic residues in the F-G loop of the globular domain, which is involved in membrane interactions. For example, this approach has been applied to CYP 17A1 by Pechurskaya et al. (10) to obtain a catalytically active protein expressed mostly in the cytosol of *E. coli*. Studies of CYP 2E1 and CYP 2B4 N-terminal helix truncation mutants have shown that the ratio of protein localized in the *E. coli* inner membrane to in the cytosol can be lowered by introducing positive charges in the flexible linker region (11). From these studies, it can be seen that the N-terminal helix is not essential for membrane association or catalytic activity, and indeed, that its structural and functional roles are unclear. Although CYPs with the N-terminal TM-helix truncated can be reconstituted as active systems with CPR and Cyt-b5, it is unknown whether the CYP TM-helix interacts with the anchoring TM-helices of CPR and Cyt-b5 in the ER. Recently, a dynamic nuclear polarization magic-angle-spinning NMR spectroscopy study of full-length CYP 2B4 and Cyt-b5 has shown inter-TM-helix interactions between the two bitopic proteins (12). However, experimental information on TM-helix interactions and orientation in the membrane (e.g., measurements of the TM-helix tilt angle with respect to the membrane plane) is scarce. The only crystal structure of a full-length membrane-binding CYP that has been resolved is for *Saccharomyces cerevisiae* lanosterol 14*α*-demethylase of the CYP51 family (Protein Data Bank (PDB): 4LXJ (13)). It consists of an amphipathic helix (residues 1–25), the TM-helix (residues 27–50), and the globular domain connected to the TM-helix by a flexible linker. The TM-helix, which is 24 residues long (37.5 Å), has a pronounced tilt angle of 55° with respect to the membrane normal (13). The primary sequence and length of the TM-helix vary in different CYPs, and most CYPs lack a preceding amphipathic helix. For example, CYP 17A1 has 508 residues and consists of a short hydrophobic N-terminal domain (residues 1–19), a long linker region (residues 20–49), and a water-soluble catalytic domain (residues 50–508) (see Fig. 1). Depending on the length of the hydrophobic sequence of the TM-helix, the distribution of amino acid residues, and the hydrophobic thickness of the membrane, the TM-helix can adopt different orientations or tilt angles in the membrane. Longer TM-helices can develop kinks to avoid hydrophobic mismatch (14).

In this study, we investigate the effects of the TM-helix on CYP-membrane interactions by comparing wild-type (wt) proteins with mutant (mt) proteins with N-terminal mutations. We use modified constructs of CYP 17A1 (pCWH17mod (9), here referred to as mtCYP17) and CYP 19A1 (NmA264R (15), here referred to as mtCYP19) in experiments to study the insertion and orientation of CYP 17A1 and CYP 19A1 in a 1-palmitoyl-2-oleoyl-sn-glycero-3-phosphocholine (POPC) Nanodisc. The mtCYP17 construct was originally made by mutating five residues in the N-terminal TM-helix region to enable expression of the protein in *E. coli* and adding a 4-histidine tag at the C-terminus (residues 509–512) for purification of the protein (9). The five substitutions introduced within the first eight N-terminal residues are W2A, E3L, V5L, L7V, and L8F. The mtCYP19 construct was made by removing the first 46 residues from the N-terminus of the wt sequence and replacing them by 11 residues (MARQSFGRGKL) to express the protein in *E. coli* (15). Here, we built models of mtCYP17 and mtCYP19 as well as wt CYP 17A1 (wtCYP17) and wt CYP 19A1 (wtCYP19) in a phospholipid bilayer using a procedure employing efficient configurational sampling with a coarse-grained (CG) representation followed by structural refinement at atomic detail. We then performed molecular dynamics (MD) simulations at both CG- and atomic-detail resolutions to investigate the effects of the mutations of the N-terminal TM-helix residues on the overall interactions and orientation in the membrane of the TM-helix region and the globular domain of the two CYPs. The results of the simulations are compared with linear dichroism measurements of the heme tilt angle for the two mt proteins and CYP 3A4 embedded in Nanodiscs. Good agreement between the experimental measurements and the simulation results is obtained, allowing us to investigate how N-terminal changes in sequence affect CYP-membrane interactions.

## Materials and Methods

### Linear dichroism experiments

#### CYP nanodiscs

Nanodiscs containing mtCYP17, mtCYP19, and CYP 3A4 were prepared and assembled according to published methods (16, 17) using the scaffold protein MSP1D1 and POPC lipids purchased from Avanti Polar Lipids (Alabaster, AL).

#### Linear dichroism measurements

A custom-built total internal reflection optical waveguide linear dichroism setup was used. The overall design consisted of a 405-nm laser diode source, a photoelastic light modulator (PEM-80; Hinds Instruments, Hillsboro, OR), a sample chamber, and a photomultiplier tube detector and integrating sphere. A beam splitter directed a portion of the incident light to a photodiode to provide a reference signal to account for variations in laser intensity. The photoelastic light modulator served as an oscillating half-wave plate, generating alternating s- and p-polarized light impinging on the optical waveguide of an annealed optically flat BK-7 slide (ArrayIt SuperClean2; ArrayIt, Sunnyvale, CA) with the light coupled in and out through BK-7 Schott glass prisms (Mainz, Germany). The sample chamber was ∼175 *μ*L in volume, constructed with separate sample and reference channels. Nanodiscs containing CYPs self-assembled onto the silicon oxide waveguide surface to form an atomically flat-oriented sample. Samples were flowed directly onto the sensor surface in a buffer of 20 mM HEPES, 150 mM NaCl, 1 mM MgCl_2_ (pH 7.3). A reference of buffer alone was taken for each sample, and samples were allowed to adsorb on the quartz for ∼2 min before taking measurements. Only the Nanodiscs bound to the surface contributed to the signal. A lock-in amplifier was set to twice the PEM operating frequency to detect the modulated linear dichroism signal, with sample and reference signals analyzed with a lock-in amplifier and analyzed in real time with LabView software. The ratio of p- and s-polarized absorbance was used to derive the heme tilt angle of the CYP protein with respect to the membrane normal according to Cropek et al. (18).

### Molecular modeling and simulation

#### Preparation of all-atom models of the proteins

The CYP 17A1 crystal structure (PDB: 3RUK) in complex with the prostate cancer drug, abiraterone, was resolved at 2.6 Å resolution after truncation to remove the N-terminal residues 1–30 (19). The crystal structure has four protein chains (A–D) in the asymmetric unit cell. Chains A and B have missing residues in the loop connecting helices H and I (residues 274–282 in chain A and 275–282 in chain B), whereas the C-terminal tail (residues 503/504–508) is absent in all four chains. Therefore, for making models of wtCYP17 and mtCYP17, the chain C structure was selected, and the missing parts in the N-terminal region (TM-helix and part of the linker) were modeled using Modeller 9v10 (20). The length of the TM-helix was predicted by the secondary structure prediction servers: PredictProtein (https://www.predictprotein.org/) (21) and PSIPRED (http://bioinf.cs.ucl.ac.uk/psipred/) (22). The TM-helix reported by Cui et al. (23) for wtCYP17 extended over residues 1–19. The N-terminal missing regions were modeled in a helical conformation by applying constraints on residues 3–19 and assuming a loop conformation for residues 20–30. The missing C-terminal residues were not included in the models. The mtCYP17 model was generated by mutating the same five residues in the N-terminal TM-helix as mutated in experiments.

The CYP 19A1 models were built using the crystal structure (PDB: 4KQ8 (24)) resolved for residues 45–496 at 3.29 Å resolution with 4-androstene-3-17-dione bound and the TM region and the C-terminal tail (residues 497–503) missing. The missing residues from the N-terminal region were modeled using Modeler 9v10. Following Park et al. (25), for wtCYP19, residues 18–38 were modeled as a TM-helix and connected to the globular domain (residues 55–496) by a linker region (residues 39–54, with residues 39–44 modeled as a loop). For mtCYP19, no TM-helix domain was predicted by secondary structure prediction servers. Therefore, the N-terminal residues (36–44) were modeled as a loop region. To facilitate comparison, the residue numbering of wtCYP19 was retained for mtCYP19. The sequences of the modeled CYPs are given in Supporting Materials and Methods.

For each of the four protein models, five different starting conformations were generated by changing the dihedral angles in the linker region (for the residue ranges specified below) to generate a diverse set of initial structures with the CYP globular domain positioned to ensure that it is outside the membrane bilayer when the protein is immersed in a bilayer (see next section). These structures were used for the construction of CG models.

#### Setup and simulation of CG systems

A similar procedure was used to generate CG models of the four proteins in a POPC bilayer in aqueous solution to that described in our previous work (26). The initial all-atom (AA) protein models were converted to MARTINI CG models using the martinize.py script (http://cgmartini.nl), and the TM-helix was immersed in a pre-equilibrated modeled CG POPC lipid bilayer consisting of 594 POPC molecules. The MARTINI version 2.2 force field with the standard water model (NPW) was used (27). The systems were neutralized by adding Cl^−^ ions (wtCYP17:6, mtCYP17:6, wtCYP19:1, and mtCYP19:7). An elastic network model was used to preserve the secondary and tertiary structure of the protein during the simulations by applying additional harmonic restraints with an elastic force constant of 500 kJ⋅mol^−1^⋅nm^−2^ and a distance cutoff range of 5–9 Å. For this purpose, the secondary structure information was provided in a DSSP file obtained from the DSSP server (http://www.cmbi.ru.nl/dssp.html).

For mtCYP17, two different CG simulation systems (mt-CG: S2–S3) were prepared with different lengths of the flexible linker that were defined by removing elastic network restraints from the linker residues 20–38 (mt-CG: S2) or 20–49 (mt-CG: S3). In the latter case, the whole linker region was set flexible. For wtCYP17 simulations (wt-CG: S1), the flexible linker length was defined as residues 20–38, as for mt-CG: S2 (Table S1). For reference, a CG model of the globular domain only (CG: S4) was also prepared for simulations. Additionally, 10 independent models of the wt (wt-CG: S5) and mt (mt-CG: S6) TM-helix (residues 1–22) alone in a membrane bilayer consisting of 594 POPC molecules were prepared by inserting the TM-helix in the bilayer using the insane.py script (28). Elastic network restraints were applied on the complete TM-helix peptide (1–22 residues).

For simulations of wtCYP19, the elastic network restraints were removed for the residues in the linker region (residues 39–54) (wtCYP19: S7), and for mtCYP19, the elastic network restraints were removed (mtCYP19: S8) or kept (mtCYP19: S9) on the N-terminal and linker regions (residues 36–54) of the protein (Table S1).

The simulation procedure was the same as described in our previous work (26). Each simulation started with a short steepest-descent energy minimization until the maximal force on a CG particle was less than 10 kJ/mol⋅nm. A 40-ns equilibration simulation at constant temperature (310 K) and pressure (1 atm) was performed in the NPT ensemble using velocity rescale v-rescale and the Berendsen procedure for pressure coupling before switching to a Parrinello-Rahman barostat method for production simulations of 8–20-*μ*s duration. A coupling constant of 12 ps was used to maintain semi-isotropic pressure coupling with a compressibility of 3.0 × 10^−5^. A time step of 20 fs was applied. The nonbonded interactions were treated with a reaction field for Coulomb interactions, and the cutoff distance for these and for van der Waals interactions was 1.1 nm.

#### Analysis of CG trajectories and back conversion to AA models

The CG simulation trajectories were analyzed for convergence and the stability of the orientations of the CYP TM-helix and globular domain in the membrane. For this purpose, we calculated the same angles and distances as defined previously to characterize the globular domain position and orientation with respect to the membrane (26, 29, 30) (Fig. 1). The angles were computed by defining the following vectors: v1, from the center of mass (CoM) of the backbone particles/atoms of the first four residues to the CoM of the last four residues of the I-helix; v2, from the CoM of the first four residues of the C-helix to the CoM of the last four residues of the F-helix; v3, the vector between the CoMs of the first and last four residues of the TM-helix and the *z* axis perpendicular to the membrane. The angle *α* was then defined as the angle between v1 and the *z* axis, and angle *β* was defined as the angle between v2 and the *z* axis. Similarly, the TM-helix tilt angle (*γ*) in the lipid membrane was defined as the angle between v3 and the *z* axis. The distances of the CoM of the globular domain, the linker region, and the F-G loop to the CoM of the lipid bilayer were monitored during the trajectories.

From the analysis of these angles and distances, a representative snapshot was selected from each set of CG simulations for a given system (5 CG simulations for the systems with full protein models, 3 for those with the globular domain only, and 10 for those with the TM-helix only), and this structure was used for back conversion to an AA model. The representative snapshot was selected to have angle and distance values within 1% of their mean value (29).

The back conversion of the POPC bilayer to an AA representation was performed as described in Cojocaru et al. (30). The back conversion of the protein was done using the scripts backward.py and initram.sh, available at the MARTINI website (http://cgmartini.nl) (31). In previous studies (26, 29), the globular domain residues of the crystal structure were superimposed on the back-mapped structure to preserve the side-chain interactions in the heme cofactor binding pocket. However, during the CG simulations of mtCYP17, the positions of the TM-helix and linker region changed such that they developed interactions with the F-G loop of the globular domain and influenced the conformation of the F-G loop and its interactions with the membrane. Here, therefore, the conformation of this and other regions making close interactions with the membrane (TM-helix, linker, A-helix, and *β*-strand1 (residues 1–76), F-G loop (residues 210–228), and *β*-strand2 (residues 378–387)) were kept the same as obtained from back conversion using the backward.py script for both wtCYP17 and mtCYP17. The conformation of the rest of the globular domain of the protein, including the heme binding pocket, was taken from the superimposed crystal structure. This procedure was also applied for the CYP 19A1 simulations and back conversion to AA model. In case of wtCYP19, the conformation of residues 1–91, 222–245, 400–412, and 464–487 and for mtCYP19, residues 36–99, 119–121, 181–184, 215–247, 400–404, and 463–489 that were making close interactions with the membrane were kept the same as obtained from back conversion using the backward.py script. The conformation of the rest of the globular domain was taken from the superimposed crystal structure.

#### All-atom molecular dynamics simulation

All-atom molecular dynamics (AAMD) simulations were performed using the AMBER ff14SB force field for the protein (32) and the LIPID14 force field for the POPC phospholipid (33) and heme parameters from Harris et al. (34) with partial atomic charges derived by density functional theory calculations. The protein-membrane system was immersed in a periodic box of TIP3P water molecules with Na^+^ and Cl^−^ ions at 150 mM ionic strength. The systems were simulated using NAMD 2.10 (35). The systems were first energy minimized with harmonic restraints, with a force constant gradually decreasing from 1000 to 0 kcal/mol⋅Å^2^ on nonhydrogen protein and lipid atoms, as described in (26). Then equilibration was performed with a time step of 1 fs in a constant surface area, pressure, and semi-isotropic ensemble for 1.5 ns with gradually decreasing harmonic restraints (from 100 to 0 kcal/mol⋅Å^2^) on nonhydrogen protein and lipid atoms. Then the system was equilibrated for a further 6 ns without harmonic restraints. During production simulations, semi-isotropic (NPAT with constant membrane area, as well as constant number of particles [N], pressure [P], and temperature [T]) (CYP 17A1) or anisotropic (NPT) (CYP 19A1, CYP 3A4) pressure coupling was used with a time step of 2 fs.

One 160-ns AAMD production simulation of mtCYP17 with the TM-helix out of the membrane core (mtCYP17-AA-OUT:1) was performed starting from a structure from trajectory mt-CG-S2:1 (see Table S2). Two AAMD simulations of mtCYP17, each of 148 ns duration, with the TM-helix in the membrane core (mtCYP17-AA-IN:1,2) were performed starting from a representative structure from trajectory mt-CG-S3:3. Two AAMD simulations of wtCYP17 (wtCYP17-AA-IN:1,2, starting from a representative structure from trajectory wt-CG-S1:4) were performed for 160 and 144 ns, respectively, for comparison with mtCYP17. One AAMD simulation (with a production run of ∼60 ns) was performed for wtCYP19 and one (with a production run of ∼50 ns) for mtCYP19. In addition, for comparison purposes, we extended the production run of CYP 3A4 that we reported previously (26) from 30 to 90 ns.

The production runs of the AAMD simulations were analyzed by computing the heme tilt angle in addition to the angles and distances computed for the CG systems. The heme tilt angle was defined as the angle between the heme plane (defined by the four nitrogen atoms coordinating the iron) and the *z* axis. Properties were computed for the complete production runs.

## Results and Discussion

### CG MD simulations

CG simulations with the MARTINI force field were performed to efficiently obtain plausible configurations of the CYP-membrane systems. The simulations were run from starting configurations with different conformations of the linker and thus different starting positions and orientations of the CYP globular domain. We first confirmed that these simulations resulted in well-defined positioning of wtCYP17 and wtCYP19 in the membrane with an N-terminal TM-helix anchor. We next investigated the effects of N-terminal mutations of the proteins on the behavior of the N-terminal linker and TM-helix region and on the final orientation and interactions of the CYP globular domain with the membrane. The systems simulated and simulations performed are listed in Table S1, and the results obtained are given in Table S3. The results for both proteins were also compared to those for our previous CG simulations of CYP 3A4 (26).

### The wtCYP17 and wtCYP19 formed stable TM-helix-membrane interactions with a converged orientation of the globular domain in the membrane

**Figure 2:**
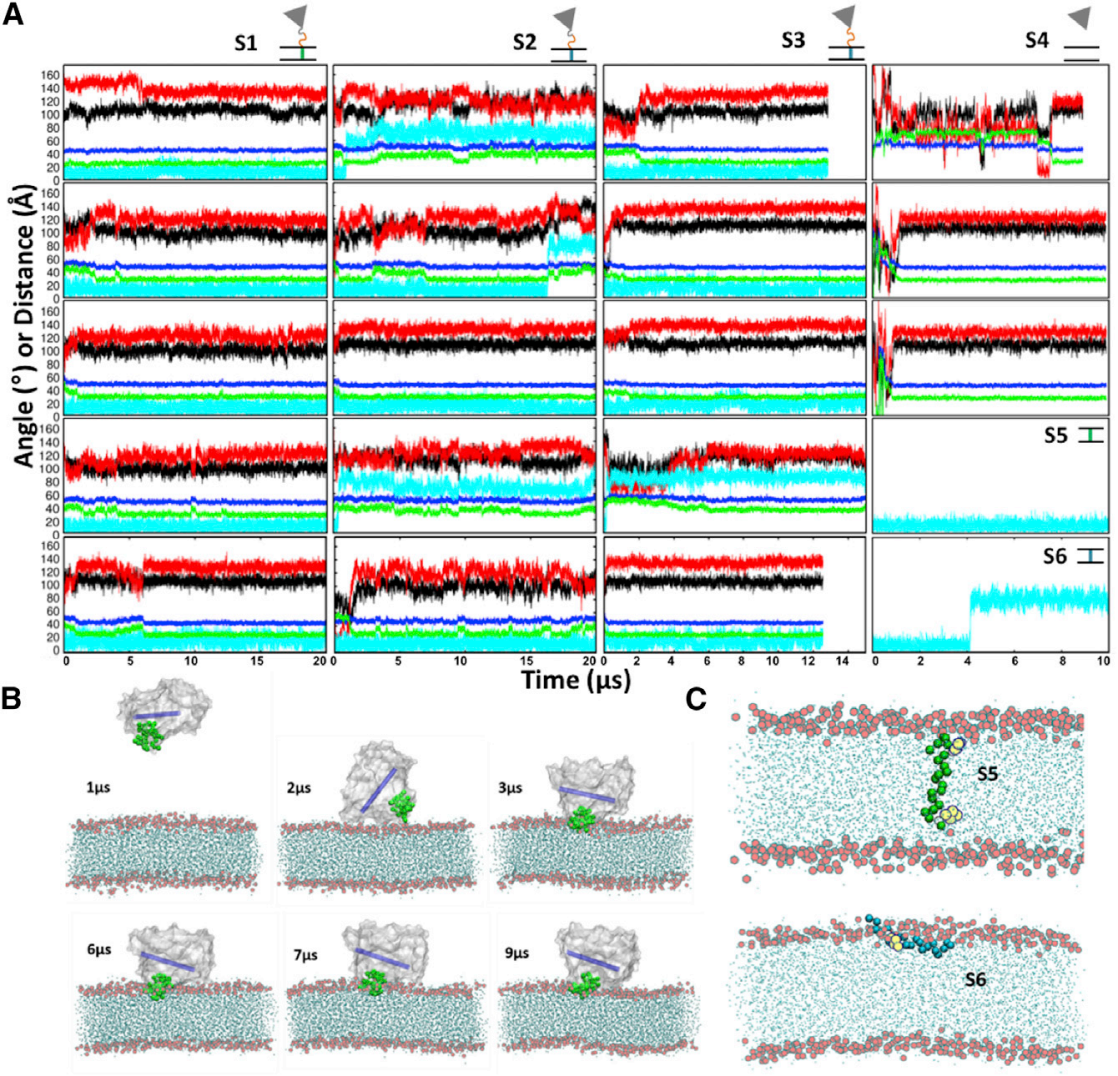
Evolution of the position and orientation of wtCYP17 and mtCYP17 with respect to the POPC bilayer during CG simulations. (*A*) Shown are plots of the angles (°) and distances (Å) characterizing the positioning of the globular domain of CYP 17A1 versus simulation time (*μ*s) for the CG simulation systems (S1–S6, shown schematically at the *top right*-*hand* corner of each panel). Plots are shown for five simulations (rows 1–5) of wtCYP17 (wt-CG-S1), two systems for mtCYP17 (mt-CG-S2 and mt-CG-S3), and three simulations (rows 1–3) of the globular-domain-only system (CG-S4). Simulations of the wild-type TM-helix (wt-TM-S5) and the mutant TM-helix (mt-TM-S6) only are shown in rows 4 and 5, respectively of column 4. The color scheme is as follows: angles (°): *α* (*black*), *β* (*red*), TM tilt angle (*cyan*); distances to the membrane CoM (Å) of the CoM of the F-G loop (*green*) and the globular domain (*blue*). (*B*) Snapshots along trajectory CG-S4:1 of the globular-domain-only system showing different orientations of the globular domain. The globular domain is shown in a silver surface representation, the F-G loop as green CG spheres, and the I-helix is represented by a blue cylinder. The POPC lipids are shown in cyan with the phosphate beads represented by red spheres. (*C*) The final snapshots from the CG simulations of the TM-helix-only systems (wt-TM-S5 and mt-TM-S6). The TM-helices are represented by green and cyan CG spheres, respectively. To see this figure in color, go online.

Five CG simulations of wtCYP17 (wt-CG-S1:1–5), each of 20-*μ*s duration, were performed. After initial reorientation, the globular domain converged to the same orientation in the five simulations, and no further large fluctuations in position were observed (Fig. 2 *A*; Table S3). The TM-helix remained inserted in the membrane, and therefore, secondary interactions established by the globular domain with the membrane were stable after the first few microseconds of the simulations. Similar behavior was observed in the five CG simulations of wtCYP19 (S7:1–5), each of 8–10-*μ*s duration, with retention of a membrane spanning position of the TM-helix and convergence of all five simulations to one orientation of the globular domain. This orientation differed from that of wtCYP17 in having a higher value of angle *β* (140 ± 6 vs. 125 ± 7°) and a closer approach of the protein to the membrane (with a globular domain CoM to membrane CoM distance of 42 ± 2 vs. 46 ± 2 Å).

### N-terminal truncation in mtCYP19 had a minimal effect on globular domain-membrane interactions

Two sets of CG simulations were run for mtCYP19, each of 8–10-*μ*s duration, with elastic network restraints either removed from the linker region (S8) or kept on the linker region (S9). During the CG MD simulations, all the orientations of mtCYP19 converged to a single configuration of the globular domain in the membrane, with the F-G-loop region interacting with the membrane that was very similar to that of wtCYP19 (Table S3). However, when the elastic network restraints were removed from the linker region of mtCYP19 (S8), the linker region stabilized parallel to the membrane in a position differing significantly from that in the crystal structure (PDB: 4KQ8). Therefore, the crystallographic position of the linker region was maintained by applying elastic network restraints on the linker region residues (S9), and a converged configuration of mtCYP19 in the membrane was selected from the S9 simulations and back converted to an AA model for AAMD simulations (see below).

### The CYP 17A1 mutant TM-helix caused unstable globular domain-membrane interactions

For the mtCYP17, five simulations (mt-CG-S2:1–5) each of 20 *μ*s duration, were carried out starting from five different initial protein conformations. As for the simulations of wtCYP17, the linker was flexible with no elastic network restraints for residues 20–38. The unstable orientation and interactions of the globular domain of mtCYP17 compared to wtCYP17 can be seen in the plots of angles and distances as a function of time (Fig. 2 *A*). This instability appears to be due to the unstable TM-helix and membrane interactions. In three (mt-CG-S2:1, 2, and 4) of the five simulations, the TM-helix drifted out of the membrane core and adopted an orientation parallel to the membrane plane as shown by the high TM-helix tilt angle (Fig. 2 *A*; Fig. S1). In only one simulation (mt-CG-S2:3) was there convergence to a final orientation corresponding to that of wtCYP17 in which the globular domain interactions with the membrane remained stable, and no fluctuations in angles and distances were observed (Table S3).

### A longer flexible linker in mtCYP17 resulted in different globular domain-membrane interactions

To ensure that the instabilities observed were not due to the elastic network restraints, five simulations were carried out with no elastic network restraints for residues 20–49 so that all residues in the linker were flexible (mt-CG-S3:1–5). Four (mt-CG-S3:1–3 and 5) out of the five simulations converged to the same orientation and remained stable for the simulation times of 12–15 *μ*s (Fig. 2 *A*). In simulation mt-CG-S3:4, the TM-helix drifted out of the membrane core and remained parallel to the membrane plane at the interface. The converged orientations for mt-CG-S3 however differ from those for mt-CG-S2 and for wtCYP17 (wt-CG-S1), having higher *α* (108 ± 9°) and *β* (134 ± 6°) angles than for the other CG systems with the TM-helix in the hydrophobic core of the bilayer (see Fig. 2 *A* and Table S3).

In all CG simulations of mtCYP17 in which the TM-helix went out of the membrane core, the interactions of the globular domain with the membrane were disrupted, and unstable secondary contacts with the membrane were formed. The distance of the CoM of the F-G loop and the CoM of the protein globular domain from the membrane CoM remained higher in cases in which the TM-helix went out of the membrane core (Fig. 2 *A*; Table S3). Apart from the TM-helix, the interactions of the CYP globular domain, especially the F-G region, and the membrane are crucial for maintaining the stable orientation of CYPs in the membrane (36, 37). The mt TM-helix not only resulted in unstable TM-helix-membrane interactions but also disrupted interactions between the globular domain and the membrane.

### The CYP 17A1 globular domain alone formed stable interactions with the membrane

To investigate the effect of removal of the linker and the TM-helix, we carried out three CG simulations (CG-S4:1–3) of only the CYP 17A1 globular domain (residues 50–502) in a bilayer. As shown in Fig. 2 *B*, the globular domain explored a large orientational and positional space before the F-G loop established contact with the membrane. The resulting F-G loop interactions with the membrane stabilized the interactions and the orientation of the globular domain above the membrane and no further changes were observed in the distances and angle plots (Fig. 2 *A*). The values of angles *α*, 105 ± 5.3°, and *β*, 123 ± 6°, in the CG-S4 simulations match well with those in the simulations of wtCYP17 (wt-CG-S1, *α* = 101 ± 7° and *β* = 125 ± 7°) and mtCYP17 (mt-CG-S2) with the TM-helix in the membrane. The *β* angle for the mt-CG-S3 system is ∼10° higher than that for wtCYP17 and the globular domain only. The simulations of mtCYP17 (mt-CG-S2 and mt-CG-S3) in which the TM-helix went out of the membrane core showed an ∼10° higher *α* angle compared to the simulations of wtCYP17 and the globular domain only.

### The wtCYP17 TM-helix spanned the membrane, whereas the mutant TM-helix lay along the membrane surface during CG simulations of only the TM-helix in the membrane

To investigate the effect of the mutations on the TM-helix alone, we next performed CG simulations of the TM-helix (residues 1–22) (i.e., 10 simulations for wtCYP17 (wt-TM-S5: 1–10) and 10 simulations for mtCYP17 (mt-TM-S6: 1–10)) in a POPC membrane. As shown in Fig. 2, *A* and *C*, the wtCYP17 TM-helix remained embedded in the lipid bilayer perpendicular to the membrane surface throughout the simulation time of 10 *μ*s, whereas the mtCYP17 TM-helix egressed from the membrane core in all 10 simulations to lie approximately parallel to the membrane surface, similarly to how an amphipathic helix would behave. In all 10 wt-TM-S5 simulations, the TM-helix spanned the membrane, like a bitopic membrane protein, with a TM-helix tilt angle of 12 ± 6°, matching the TM-helix tilt angle observed for the full-length wtCYP17 of 13 ± 6°.

### Key features of the sequence determine the orientation and position of the CYP 17A1 TM-helix in the membrane

**Figure 3:**
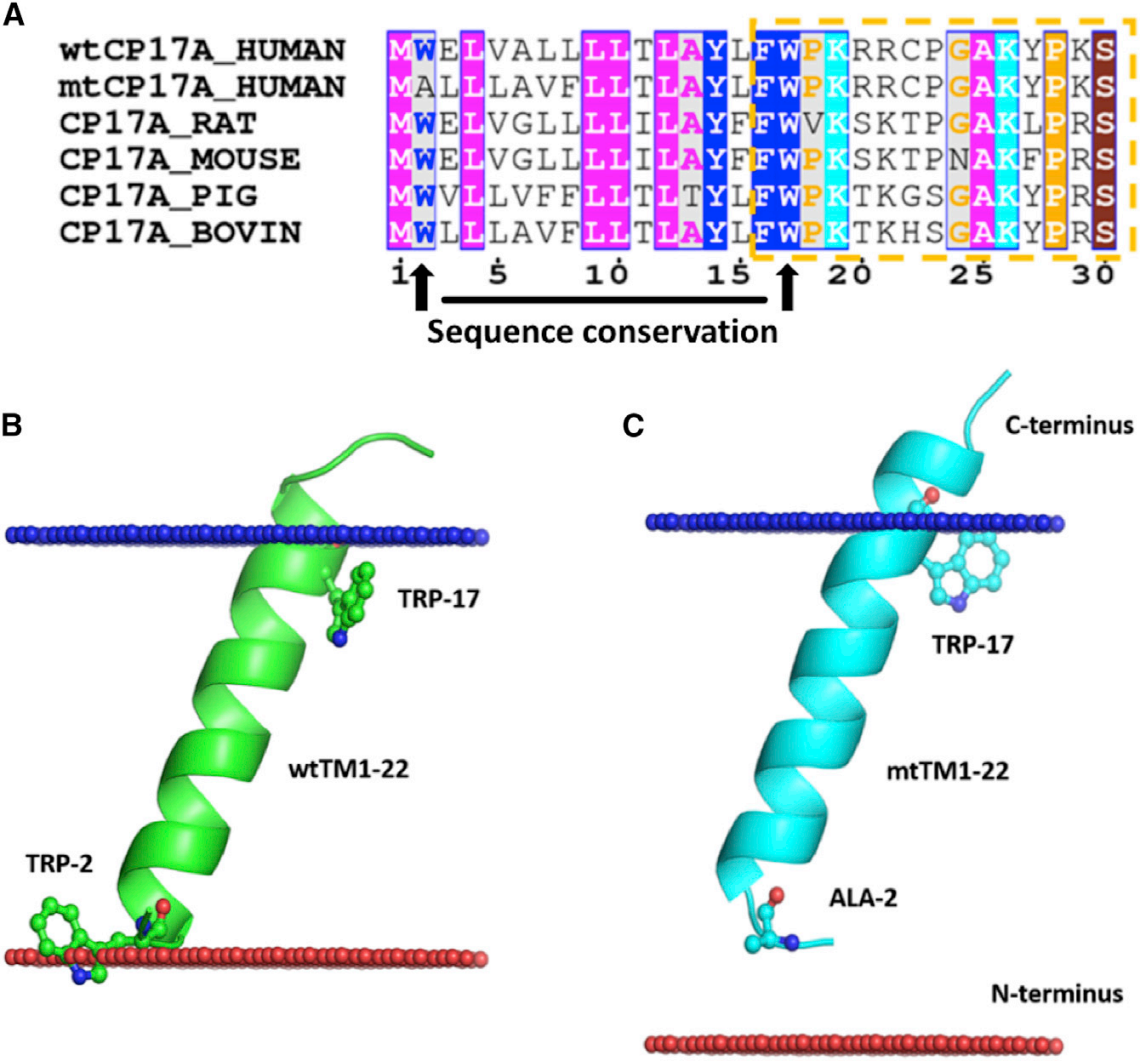
(*A*) Multiple sequence alignment of the first 30 residues of six CYP 17 sequences: human wtCYP17 and mtCYP17, rat, mouse, pig, and bovine. The color code differentiates the residues based on their physicochemical properties and conservation. The figure was generated using ESPript 3.0 (57). The orientation and position predicted by the PPM server for (*B*) wtCYP17 (*green*) and (*C*) mtCYP17 (*cyan*) residues 1–22 (cartoon representation with residues 2 and 17 in stick representation) are shown. The hydrophobic boundaries of the membrane are shown by blue and red spheres. To see this figure in color, go online.

Compared to the TM-helices in other CYPs, such as CYP 19A1 (21 residues) and CYP 3A4 (24 residues), CYP 17A1 has a short TM-helix (17 residues). On the other hand, it has a longer linker region (residues 20–49) connecting the globular domain (residues 50–502) with the TM-helix (e.g., the CYP 19A1 linker has only 17 residues). Shorter peptides or TM-helices can compensate the hydrophobic mismatch with the membrane by forming aggregates, undergoing a conformational change in the backbone residues or aligning parallel to the *z* axis so as to avoid the insertion of any charged residues in the hydrophobic core (38). Consequently, a lower TM-helix tilt angle is observed for shorter TM-helices. Thus, the combined effect of the negative mismatch due to the short length of the CYP 17A1 TM-helix and the N-terminal mutations contributed to the expulsion of the mt TM-helix from the hydrophobic core of the membrane that is observed in the simulations (Fig. 2 *C*). Although short in length, the wtCYP17 TM-helix is flanked by polar aromatic and charged residues at both ends of the TM-helix: W2 and E3 on the N-terminal end and Y14 and W17 followed by a patch of positively charged residues 19KRR21 at the C-terminal end (Fig. 3), which result in the stable interactions and orientation in the membrane. Furthermore, a multiple-sequence alignment of the first 30 residues of human wtCYP17 and mtCYP17 as well as CYP 17 sequences from various mammals shows that the aromatic residues, W2, Y14, F16 and W17, are conserved, see Fig. 2.

Tryptophan (W) and Tyrosine (Y) residues in membrane proteins act as anchoring residues and are generally located between the polar headgroup and hydrophobic core of the lipid bilayer in the glycerol region of the lipid bilayer (14). The presence of W2, W17, and charged residues on either side of the TM-helix of wtCYP17 stabilizes the orientation of the TM-helix in the membrane. Thus, the wtCYP17 TM-helix showed bitopic characteristics during CG simulations of the full protein (wt-CG-S1) and of the TM-helix only (wt-TM-S5) and fully spanned the hydrophobic core of the membrane. On the other hand, the W2A and E3L mutations disrupted the balance and resulted in the formation of a short helix with no charged residues at the N-terminus. Despite its greater hydrophobicity, resulting in greater predicted ER membrane insertion propensity ((39); http://dgpred.cbr.su.se/index.php?p=TMpred), the mtCYP17 TM-helix went out of the membrane core during the simulations.

The position of the TM-helix (residues 1–22) in the membrane was predicted for both wtCYP17 and mtCYP17 using the Position of the Protein in Membrane (PPM) prediction server, (http://opm.phar.umich.edu/server.php) (40). The orientation and position of the two TM-helices differ from each other in the insertion depth and TM-helix tilt angle. The TM-helix tilt angle predicted by the PPM server for mtCYP17 was 18 ± 12°, which is close to the mtCYP17 TM-helix tilt angle observed in CG simulations (mt-CG-S2 and mt-CG-S3). A lower TM-helix tilt angle was predicted by the PPM server for the wtCYP17 TM-helix (13 ± 6°), which is consistent with the TM-helix tilt angle observed in CG simulations (wt-CG-S1) of 13 ± 7° (Table S3) and AAMD simulations of 13 ± 8° and 15 ± 7°, respectively (see next section). A similar TM-helix tilt angle 12 ± 6° was observed in the simulations of the wtCYP17 TM-helix only (wt-TM-S5). Besides the TM-helix tilt angle, the predicted insertion depth of the wtCYP17 TM-helix and the mtCYP17 TM-helix also differs. As shown in Fig. 3, the wtCYP17 TM-helix is predicted by the PPM server to span the hydrophobic core of the membrane like a bitopic TM-helix, whereas the mtCYP17 TM-helix shows monotopic TM-helix characteristics. Furthermore, the PredictProtein server (21) did not predict any TM-helix for the full mtCYP17 sequence, whereas it predicted a TM-helix for residues 6–19 in wtCYP17.

### Different positions of the linker were observed for wtCYP17 and mtCYP17

**Figure 4:**
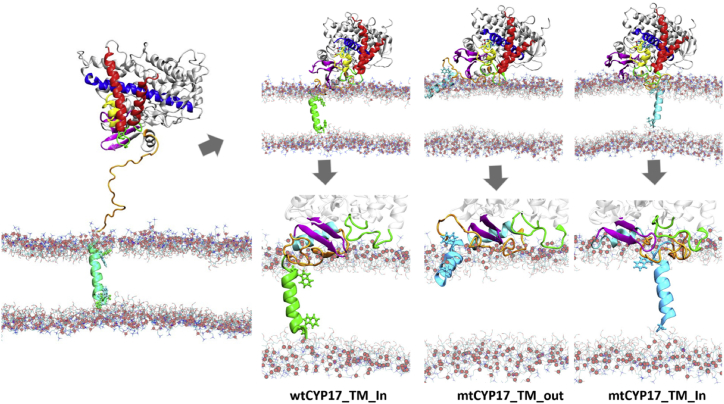
Positioning of the TM-helix, linker, and the globular domain before and after CG simulations of CYP 17A1. Left: initial representative superimposed frames for the CG simulations of the wtCYP17 (*green* TM-helix) and the mtCYP17 (*cyan* TM-helix) are shown. Right: structures from the final snapshots of three CG simulations are shown for the full system (*above*), and with a close-up showing the position of the TM-helix (*below*) (wtCYP17: *green*; mtCYP17: *cyan*) and the arrangement of the linker (*orange*) with respect to *β*-strands 1–4 (*magenta*) and the F-G loop (*green*). Differences in the linker with respect to the F-G loop are observed. The linker in wtCYP17 (*left*) lies on the proximal side and develops contact with *β*-strands 1–2. A similar position of the linker is observed for mtCYP17 when the TM-helix leaves the bilayer core (*middle*), whereas the linker is trapped and forms polar contacts with the F-G loop in mtCYP17 when the TM-helix remains spanning the bilayer (*right*). This position of the linker would hinder the access of substrates from the membrane to the active site. All structures are shown after back conversion to atomic detail. To see this figure in color, go online.

The position and conformation of the linker region were affected by the TM-helix sequence and by the position of the TM-helix in the membrane. The linker in CYP 17A1 is rather long at 32 residues compared with the linker length in, for example, CYP 19A1 (17 residues) and CYP 3A4 (22 residues). Two different lengths of flexible linkers were defined by elastic network restraints in the mtCYP17 simulations to observe whether linker length affected the interactions of the globular domain of mtCYP17 in the membrane. During the CG simulations with the longer flexible linker (mt-CG-S3), the linker in mtCYP17 deviated from the wtCYP17 linker position and developed polar interactions with the F-G region in mtCYP17 (see Fig. 4). In CG simulations of mtCYP17 in which the TM-helix remained in the membrane, we observed conformational changes in the TM-helix backbone residues along with the unwinding of the helix to resolve the hydrophobic mismatch.

The linker conformation in simulations of wtCYP17 matched with the conformation of the part of the linker observed in the crystal structure and did not show interactions with the F-G loop (Fig. S2). In the simulations of mtCYP17, a similar position of the linker was observed when the TM-helix left the bilayer core (Fig. 4), but when the TM-helix spanned the bilayer, the F-G loop region became blocked by the linker, indicating that it may interfere with substrate access via tunnel 2d or 2f from the membrane to the active site (Figs. 4 and S3). The 2d tunnel egresses the globular domain between the N-terminus and helices a/A′ and A, whereas tunnel 2f egresses between the F′-helix/F-G loop and the *β*5 sheet (41). Tunnel 2f in CYP 17A1 is lined by aromatic gating residues (W220 and F224) (42), which were displaced by the linker in simulations of mtCYP17. The side chain of F224 flipped outward compared to the crystal structure and remained in this position (see Fig. S4).

#### AAMD simulations

The AAMD simulations were performed to observe CYP-membrane interactions in atomic detail by starting the simulations from representative frames from the CG simulations (see Table S2). For CYP 17A1, three different starting structures were used: wtCYP17 and mtCYP17 with the TM-helix in the bilayer core (IN) and mtCYP17 with the TM-helix at the bilayer interface (OUT). AAMD simulations for each protein with the TM-helix IN were performed twice with different initial velocities. Thus, five trajectories were generated for CYP 17A1: wtCYP17-AA-IN:1–2, mtCYP17-AA-OUT:1, and mtCYP17-AA-IN:1–2. Two trajectories were generated for CYP19A1: wtCYP19-AA and mtCYP19-AA, starting with the representative structure selected from the S9 simulations. The trajectories were analyzed to characterize the CYP-membrane interactions by computing the membrane insertion depth and orientation angles of the globular domain as for the CG simulations. Additionally, for AAMD simulations, the heme tilt angle was computed and compared with the heme tilt angles measured by linear dichroism.

### AAMD simulations showed stable arrangements of the CYP globular domain with respect to the bilayer after slight repositioning

**Figure 5:**
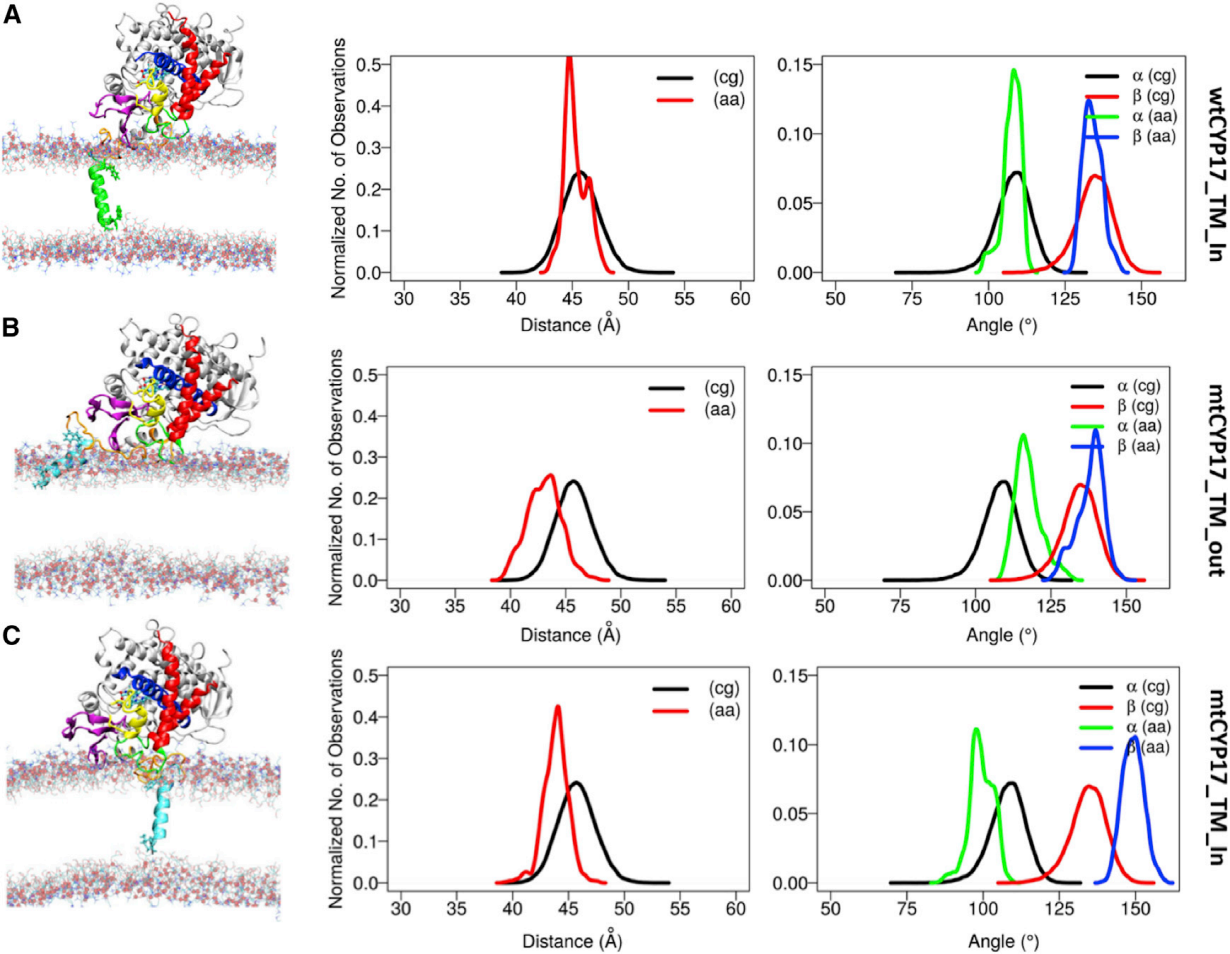
Insertion depth and orientation of the globular domain in AAMD simulations of wtCYP17 and mtCYP17 compared to the corresponding CG simulations for (*A*) wtCYP17-AA-IN:1, (*B*) mtCYP17-AA-OUT:1, and (*C*) mtCYP17-AA-IN:1. The last frame of each simulation is shown on the left, and the population distribution plots of angles and distances in CG and AAMD simulations are shown on the right. The proteins are shown in cartoon representation with wtCYP17 with a green TM-helix and mtCYP17 with a cyan TM-helix. The globular domain is colored as follows: the linker is in orange, *β*-strands 1–4 are in magenta, F- and G-helices are in red, I-helix is in blue, B′-C′ loop is in yellow, and the F-G loop is in green. To see this figure in color, go online.

As shown in the plots of the distributions of angles and distances over simulation time (Fig. 5; Table 1), compared to the CG simulations, the *α* and *β* angles defining the globular domain orientation shifted slightly, whereas the globular domain on average inserted further into the membrane. The readjustments in the position of the globular domain occurred at the beginning of the simulations, mostly within the first 20 ns (Fig. S5).

**Table 1:** Angles and Distances Characterizing the Protein Orientation and Insertion Depth in the Membrane in the AAMD Simulations of Wild-Type and Mutant CYP 17A1, CYP 19A1, and CYP 3A4

Protein	Simulation	CoM-CoM Distances (Å)			Angles (°)				
		Globular Domain	Linker	F-G Loop	*α*	*β*	TM-Helix Tilt	Heme Tilt	
								Computed^a^	Measured
wtCYP17	*wt-CG-S1:1–5*	*46 ± 2*	*20 ± 2*	*27 ± 2*	*101 ± 7*	*125 ± 7*	*13 ± 7*	*44.9*	NA
	wtCYP17-AA-IN:1	46 ± 2	21 ± 2	26 ± 2	106 ± 8	126 ± 5	12 ± 7	40 ± 5	
	wtCYP17-AA-IN:2	45 ± 2	21 ± 2	25 ± 2	107 ± 5	137 ± 7	18 ± 7	45 ± 8	

mtCYP17	*mt-CG-S2:1*^b^	*45*	*24*	*27*	*106*	*136*	*61*	*55.7*	62.1 ± 0.9
	mtCYP17-AA-OUT:1	43 ± 2	23 ± 2	23 ± 2	112 ± 4	138 ± 4	63 ± 10	50 ± 6	
	*mt-CG-S3:1-3,5*	*46 ± 2*	*22 ± 2*	*28 ± 2*	*108 ± 6*	*134 ± 6*	*15 ± 8*	*48.8*	
	mtCYP17-AA-IN:1	44 ± 2	21 ± 2	24 ± 2	105 ± 7	136 ± 4	20 ± 5	59 ± 6	
	mtCYP17-AA-IN:2	45 ± 2	22 ± 1	26 ± 2	110 ± 6	147 ± 5	24 ± 7	54 ± 5	

wtCYP19	*CG* (*S7*)	*42 ± 2*	*22 ± 2*	*23 ± 2*	*104 ± 5*	*140 ± 6*	*16 ± 8*	*54.8*	NA
	AA	42 ± 2	21 ± 2	23 ± 2	102 ± 5	131 ± 4	19 ± 6	50 ± 5	

mtCYP19	*CG* (*S8*)	*45 ± 1*	*24 ± 2*	*26 ± 2*	*104 ± 5*	*152 ± 5*	*NA*	*NA*	57.5 ± 0.8
	*CG* (*S9*)	*44 ± 1*	*35 ± 5*	*26 ± 2*	*107 ± 6*	*150 ± 5*	*NA*	*59.3*	
	AA	42 ± 1	24 ± 2	24 ± 2	106 ± 4	142 ± 5	NA	54 ± 5	

CYP 3A4	*CG* (*S10*)^c^	*42 ± 2*	*25 ± 4*	*25 ± 4*	*65 ± 7*	*139 ± 7*	*31 ± 7*	*53.7*	60.0 ± 1.6
	AA^c^	42 ± 2	*17 ± 2*	*17 ± 2*	70 *± 10*	144 ± 5	49 ± 4	58 ± 5	

Values of the means and SDs were computed over the production runs (see Tables S1 and S2). For comparison, the values for CG simulations are shown in italics, and the experimentally measured heme tilt angles are given in the last column. NA, not applicable.

a For the CG simulations, the heme tilt angle was computed after conversion of the representative CG frame to an AA representation.

b The values are shown for the selected frame from the CG simulation with the TM-helix OUT rather than the full simulation as the TM-helix transitioned to an OUT position at the bilayer interface during the simulation.

c Values for CG simulations from (26). The AAMD simulation reported in this article was extended from 30 to 90 ns to compute the values for the AA model.

The structure of the CYP globular domain remained stable throughout the AAMD simulations. The average root mean-square deviation of the C-*α* atoms of the globular domain (residues 50–502) of CYP 17A1 with respect to the minimized structure was equal to or below 3 Å (Fig. S6), and for CYP 19A1, the average C-*α* atom root mean-square deviation was below 2.0 Å. The B-factor values computed from AAMD simulations for CYP 17A1 showed a similar overall pattern of fluctuations to the B-factors in the crystal structure. Higher B-factor values were observed mainly in the TM-helix, linker regions, and the different loops in contact with the membrane or the aqueous solvent. Compared to wtCYP17 simulations, lower B-factors were seen in mtCYP17 in which the linker is trapped in the F-G loop region.

#### CYP heme tilt angles from computations and experiments are consistent

The CYPs were incorporated in Nanodiscs, and the values of the heme tilt angle measured by linear dichroism are given in Table 1. A number of functional studies have demonstrated the catalytic competency of Nanodisc-incorporated CYPs (16, 43, 44, 45). The structural properties of CYPs incorporated in Nanodiscs have previously been investigated by a variety of experimental modalities, including small-angle x-ray scattering, solution and solid-state NMR (ssNMR), and atomic force microscopy (46, 47, 48). These studies all indicate that the N-terminal membrane anchor is embedded in the lipid bilayer of the Nanodisc.

In our previous work on multiscale simulations of CYP 3A4 in the membrane, we reported a heme tilt angle of 53.7° from CG simulation, which decreased to 52.7 ± 3° after running 30-ns AAMD simulations (26). On extending the simulation to ∼90 ns here, the heme tilt angle increased to 61.0 ± 3.8°, which corresponds well with the value of the heme tilt angle of CYP 3A4 in a POPC Nanodisc measured here (60.0 ± 1.6°) and reported previously (49). The heme tilt angle observed in this simulation differs from the predicted orientations of CYP 3A4 in the Orientations of Proteins in Membranes (OPM) database for recently published structures of CYP 3A4 in apo form (PDB: 1TQN) and bound to midazolam (PDB: 5TE8) with heme tilt angles of 80.3 and 77.5°, respectively. Furthermore, our simulations of mtCYP19 in a membrane resulted in a computed heme tilt angle of 54 ± 5°, close to the experimentally determined angle for mtCYP19 in a Nanodisc of 57.5 ± 0.8° (see Table 1), whereas the heme tilt angle for the predicted orientation in the OPM database is 64.3°.

The values of the heme tilt angle in simulations of mtCYP17 with the TM-helix in the membrane core (mtCYP17-AA-IN:1 and 2) were 59 ± 6° and 54 ± 5°, close to the heme tilt angle observed in our experiments for mtCYP17 in a Nanodisc of 62.1 ± 0.9°. The heme tilt angle in the simulation of mtCYP17 with the helix out of the membrane core (mtCYP17-AA-OUT:1) was somewhat lower at 50 ± 6°. In contrast to mtCYP17, in simulations of wtCYP17 (wtCYP17-AA-IN:1–2), the heme tilt angle was lower at 40 ± 5° and 45 ± 8°. These values of the heme tilt angle in wtCYP17 are consistent with those observed in simulations of wtCYP17 by Cui et al. (42), which varied between 40 and 67°. The values of the heme tilt angle for predicted orientation in the OPM database are 65° (PDB: 3SWZ) and 33° (PDB: 5UYZ). The range of values of the heme tilt angle suggests that the CYP 17A1 globular domain can adopt various orientations in the membrane and that because of its sequence, its orientation is particularly sensitive to additional or external factors, such as N-terminal mutation.

### AAMD simulations showed greater variations of the position of the TM-helix in mtCYP17 than in wtCYP17

The TM-helix tilt angle was higher and showed a greater range in the mtCYP17 simulations than in the wtCYP17 simulations. For the latter, the tilt angles were 12 ± 7° and 18 ± 7° (Table 1), corresponding well to the previously reported low tilt angles observed in short TM-helices in AA simulation studies (50), the tilt angle predicted by the PPM server (see above), and the TM-helix tilt angle measured by ssNMR spectroscopy study for full-length rabbit CYP 2B4 of 17 ± 3° (41), with a 19-residue-long TM-helix. In the presence of polar aromatic and charged flanking residues on both sides of the TM-helix, the wt TM-helix remained stable in the membrane (see Fig. S5). However, because of the short length (17 residues) of the TM-helix and the negative hydrophobic mismatch effect, curvature of the membrane was observed near the N-terminal end of the TM-helix, and this was more pronounced in mtCYP17 than in wtCYP17. In contrast, for CYP 3A4 with its longer TM-helix of 24 residues (residues 3–26), the TM-helix tilt angle of 31 ± 7° in CG MD simulations increased to 49 ± 4° in the AAMD simulations, and the TM-helix bent on the N-terminal side. The higher TM-helix tilt angle observed in CYP 3A4 is closer to the tilt angle of the 24-residue-long TM-helix in the crystal structure of full-length CYP 51 of 55°.

### Comparison of mtCYP17 and wtCYP17 shows how the positioning of the globular domain, flexible linker, and TM-helix may affect substrate access tunnels in mtCYP17

**Figure 6:**
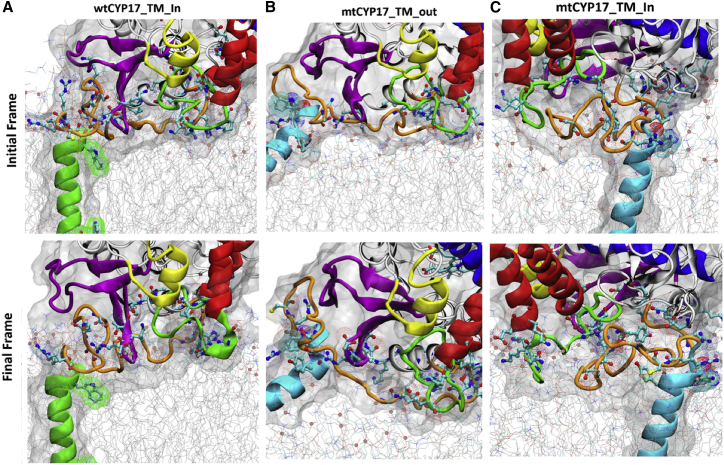
Protein-membrane interactions in the AAMD simulations. The initial and last frames are shown from simulations (*A*) wtCYP17-AA-IN:1, (*B*) mtCYP17-AA-OUT:1, and (*C*) mtCYP17-AA-IN:1, showing the position of the linker (*orange*) with respect to the F-G loop (*green*) and *β*-strands 1–4 (*magenta*). The polar and charged residues within 5 Å of the phosphate atoms (*light-red spheres*) of the POPC membrane are shown in cyan carbon ball-and-stick representation. To see this figure in color, go online.

The different positions of the linker with respect to the A-helix and the F-G loop in mtCYP17 and wtCYP17 are highlighted in Fig. 6. Compared to wtCYP17, the *α* and *β* angles are mostly higher for mtCYP17, resulting in an increased heme tilt angle (50–59° vs. 40–45°). Consequently, the distal side of mtCYP17 is more tilted toward the membrane, and the F-G loop is more inserted in the membrane than for wtCYP17, which is also evident from the distances of the F-G loop and the globular domain from the membrane. Thus, the positioning of the entrances to substrate access channels with respect to the bilayer differs in mtCYP17 from wtCYP17. For CYP 19A1, mtCYP19 also has a slightly higher *β*-angle than wtCYP19 (142.0 vs. 130.9°), resulting in a higher heme tilt angle (54 ± 5° vs. 50 ± 5°).

## Conclusions

Our simulations of three CYPs in a phospholipid bilayer show good agreement with linear dichroism measurements of the heme tilt angles of the CYPs incorporated in Nanodiscs. The structures and motions of the CYP globular domains are consistent with crystallographic data. The development of force fields and protocols for the simulation of membranes and for the simulation of heterogeneous systems, such as those studied here, consisting of protein, lipid, cofactors, and solvent molecules and ions, is an active area of research. For example, sensitivity of the tilt and kink angle distributions of helix A of bacteriorhodopsin to system size (84 vs. 128 POPC molecules) was observed in MD simulations (51). Sampling of the configurational space of CYP-membrane systems is challenging and, for this purpose, we have chosen to combine running longer CG replicas, for which convergence can be more readily monitored both within and between replicas, with shorter AA replicas starting from representative coordinates generated in CG simulations. Encouragingly, a recent critical comparison of force fields for simulating diverse protein-membrane systems showed rather good performance for protein-lipid interactions at the membrane interface for the atomic detail AMBER ff14sb/LIPID 14 force field combination that we have used in this work (52). Moreover, the similarity of the CYP-membrane arrangements from our CG and AA simulations as well as the agreement with experimental observations suggests that our simulations can be used to examine the dependence of CYP positioning in the membrane on protein sequence.

Our simulations indicate that because of sequence differences, the orientation and membrane interactions of a CYP in a phospholipid bilayer vary between CYPs and show varying degrees of sensitivity to mutation of their N-terminal anchor and linker sequences. We find that the orientation of the CYP 17A1 globular domain is particularly sensitive to N-terminal sequence alterations. The mutation of two residues, W2A and E3L, in a construct, mtCYP17, introduced for expressing the protein in *E. coli*., removes an anchor at the end of the TM-helix and leads to unstable interactions of the TM-helix in the membrane. In several CG simulations of mtCYP17, the TM-helix egressed from the membrane interior and lay at the membrane interface, almost parallel to the membrane plane. Expulsion of the TM-helix, in turn, resulted in unstable orientations of the globular domain with respect to the membrane. In the simulations of mtCYP17 in which the TM-helix remained in the membrane, it was stabilized because of interactions between the polar and charged linker residues (19, 20, 21, 22, 23, 24, 25, 26, 27, 28, 29, 30) and the F-G loop region. Additionally, in some of the simulations, the mt TM-helix was retained in the membrane by distortion of the TM-helix backbone to extend it, leading to partial unwinding of the TM-helix in the membrane.

W2 is conserved in different species of CYP 17A1. The substitutions of polar/charged membrane-anchoring residues (W2A and E3L) at the N-terminus in mtCYP17 result in completely hydrophobic residues at the N-terminal end and polar aromatic and charged (19KRR21) residues at the C-terminal end of the TM-helix. The lack of N-terminal anchoring residues results in the tendency for the TM-helix to leave the membrane core, resulting in a monotopic membrane protein and increased TM-helix tilt angle. The unstable TM-helix-membrane interactions in mtCYP17 also influenced the orientation of the globular domain in the membrane, as shown by differences in the heme tilt angles of mtCYP17 (59 ± 6°) and wtCYP17 (40 ± 5°). The linker position, membrane interactions, and degree of insertion also differ between the two CYPs. In mtCYP17, when the TM-helix is inside the membrane core, the linker is trapped in a position where it can obstruct the opening of the substrate entrance tunnels 2f/2d to the membrane, possibly affecting catalytic activity.

To perform its lyase activity, CYP 17A1 requires the allosteric redox partner Cyt-b5, which is colocalized with CYP 17A1 in the adrenal zona reticularis and gonads, and the stimulation of this activity is manifested in Nanodiscs in reconstituted systems of the CYP, CPR, and Cyt-b5 (53). Dynamic nuclear polarization magic-angle-spinning ssNMR spectroscopy showed that a full-length CYP 2B4 and Cyt-b5 have similar TM-helix tilt angles, 17 ± 3° and 15 ± 3°, respectively, and develop inter-TM-helix interactions (12, 54). Such TM-helix interactions could be crucial for forming stable interactions between the globular domains of CYP 17A1, with its short TM-helix, and Cyt-b5, as it has been shown that C-terminal truncation of the TM region of Cyt-b5 abolishes stimulation of the lyase activity of mtCYP17 (55). Moreover, mutations in the TM-helix of CYP 17A1 that reduce the stability of the TM-helix in the core of the ER membrane could influence CYP 17A1-Cyt-b5 TM-helix interactions and thereby reduce lyase activity, as observed experimentally (9). On the other hand, there is no evidence that the TM-helix of the NADPH-redox partner, CPR, makes interactions with CYP TM-helices, although a TM-helix tilt angle of 13 ± 2° has been measured for CPR (56). Thus, the N-terminal mutations in mtCYP17 may have little influence on binding of CPR to the CYP 17A1 globular domain and therefore, have no effect on the hydroxylase activity of CYP 17A1, as suggested by experiments (9).

It has been shown for a number of membrane-bound CYPs that their globular domains alone can bind to membranes and are catalytically competent in vitro but not in mammalian cells (8). The N-terminal sequence is important for translocation to and retention in the ER membrane. Moreover, the TM-helix provides an additional stabilization of the structure in the membrane that may play a role in modulating the interactions with the redox partners, Cyt-b5 and CPR, as well as homo- and hetero-oligomerization of CYPs. Our results show that the stability of the CYP-membrane interactions varies among CYPs and that some CYPs, such as CYP 17A1, may be particularly sensitive to variations in the N-terminal sequence, which may affect their function through interactions of the globular domain, the flexible linker, and the TM-helix in the membrane. Such effects of small variations in the sequences of anchoring TM-helices may also be pertinent to other membrane proteins that are anchored by a single TM-helix.

## Author Contributions

G.M., P.P.N., S.G.S., and R.C.W. designed the research. G.M. and P.P.N. performed computations, and M.G., P.P.N., N.J.B., and R.C.W. analyzed the computational data. T.J.C. performed the experiments, and T.J.C., M.C.G., and S.G.S. analyzed the experimental data. G.M., P.P.N., and R.C.W. wrote the manuscript with contributions from all authors.

## Supporting Material

Document S1. Supporting Materials and Methods, Figs. S1–S6, and Tables S1–S3

Document S2. Article plus Supporting Material
