# Supplementary material for: Influence of Transmembrane Helix Mutations on Cytochrome P450-Membrane Interactions and Function
Source: Biophys J. 2019 Jan 3;116(3):419–32. doi: 10.1016/j.bpj.2018.12.014 (PMC6369400; doi:10.1016/j.bpj.2018.12.014)
Supplement: Document S1. Supporting Materials and Methods, Figs. S1–S6, and Tables S1–S3 [file mmc1.pdf]

Biophysical Journal, Volume *116*

Supplemental Information

Influence of Transmembrane Helix Mutations on Cytochrome P450-Membrane Interactions and Function

Ghulam Mustafa, Prajwal P. Nandekar, Tyler J. Camp, Neil J. Bruce, Michael C. Gregory, Stephen G. Sligar, and Rebecca C. Wade

Supporting Methods: CYP 17A1 and CYP 19A1 sequences modelled and simulated*****

1) Human CYP 17A1 wild type (wtCYP17) Uniprot Id: P05093

```
MWELVALLLLTLAYLFWPKRRCPGAKYPKSLLSLPLVGSLPFLPRHGHMHNNFFKLQKKY
GPIYSVRMGTKTTVIVGHHQLAKEVLIKKGKDFSGRPQMATLDIASNNRKGIAFADSGAH
WQLHRRLAMATFALFKDGDQKLEKIICQEISTLCDMLATHNGQSIDISFPVFVAVTNVIS
LICFNTSYKNGDPELNVIQNYNEGIIDNLSKDSLVDLVPWLKIFPNKTLEKLKSHVKIRN
DLLNKILENYKEKFRSDSITNMLDTLMQAKMNSDNGNAGPDQDSELLSDNHILTTIGDIF
GAGVETTTSVVKWTLAFLLHNPQVKKKLYEEIDQNVGFSRTPTISDRNRLLLLEATIREV
LRLRPVAPMLIPHKANVDSSIGEFAVDKGTEVIINLWALHHNEKEWHQPDQFMPERFLNP
AGTQLISPSVSYLPFGAGPRSCIGEILARQELFLIMAWLLQRFDLEVPDDGQLPSLEGIP
KVVFLIDSFKVKIKVRQAWREA
```

2) Human CYP 17A1 mutated (mtCYP17)

```
MALLLAVFLLTLAYLFWPKRRCPGAKYPKSLLSLPLVGSLPFLPRHGHMHNNFFKLQKKY
GPIYSVRMGTKTTVIVGHHQLAKEVLIKKGKDFSGRPQMATLDIASNNRKGIAFADSGAH
WQLHRRLAMATFALFKDGDQKLEKIICQEISTLCDMLATHNGQSIDISFPVFVAVTNVIS
LICFNTSYKNGDPELNVIQNYNEGIIDNLSKDSLVDLVPWLKIFPNKTLEKLKSHVKIRN
DLLNKILENYKEKFRSDSITNMLDTLMQAKMNSDNGNAGPDQDSELLSDNHILTTIGDIF
GAGVETTTSVVKWTLAFLLHNPQVKKKLYEEIDQNVGFSRTPTISDRNRLLLLEATIREV
LRLRPVAPMLIPHKANVDSSIGEFAVDKGTEVIINLWALHHNEKEWHQPDQFMPERFLNP
AGTQLISPSVSYLPFGAGPRSCIGEILARQELFLIMAWLLQRFDLEVPDDGQLPSLEGIP
KVVFLIDSFKVKIKVRQAWREA
```

3) Human CYP 19A1 wild type (wtCYP19) Uniprot Id: P11511

```
MVLEMLNPIHYNITSIVPEAMPAATMPVLLLTGLFLLVWNYEGTSSIPGPGYCMGIGPLI
SHGRFLWMGIGSACNYYNRVYGEFMRVWISGEETLIISKSSSMFHIMKHNHYSSRFGSKL
GLQCIGMHEKGIIFNNNPELWKTTRPFFMKALSGPGLVRMVTVCAESLKTHLDRLEEVTN
ESGYVDVLTLLRRVMLDTSNTLFLRIPLDESAIVVKIQGYFDAWQALLIKPDIFFKISWL
YKKYEKSVKDLKDAIEVLIAEKRRRISTEEKLEECMDFATELILAEKRGDLTRENVNQCI
LEMLIAAPDTMSVSLFFMLFLIAKHPNVEEAIIKEIQTVIGERDIKIDDIQKLKVMENFI
YESMRYQPVVDLVMRKALEDDVIDGYPVKKGTNIILNIGRMHRLEFFPKPNEFTLENFAK
NVPYRYFQPFGFGPRGCAGKYIAMVMMKAILVTLLRRFHVKTLQGQCVESIQKIHDLSLH
PDETKNMLEMIFTPRN
```

4) Human CYP 19A1 mutated (mtCYP19)

```
------------------------------------MARQSFGRGKLIPGPGYCMGIGPLI
SHGRFLWMGIGSACNYYNRVYGEFMRVWISGEETLIISKSSSMFHIMKHNHYSSRFGSKL
GLQCIGMHEKGIIFNNNPELWKTTRPFFMKALSGPGLVRMVTVCAESLKTHLDRLEEVTN
ESGYVDVLTLLRRVMLDTSNTLFLRIPLDESAIVVKIQGYFDAWQALLIKPDIFFKISWL
YKKYEKSVKDLKDAIEVLIAEKRRRISTEEKLEECMDFATELILAEKRGDLTRENVNQCI
LEMLIAAPDTMSVSLFFMLFLIAKHPNVEEAIIKEIQTVIGERDIKIDDIQKLKVMENFI
YESMRYQPVVDLVMRKALEDDVIDGYPVKKGTNIILNIGRMHRLEFFPKPNEFTLENFAK
NVPYRYFQPFGFGPRGCAGKYIAMVMMKAILVTLLRRFHVKTLQGQCVESIQKIHDLSLH
PDETKNMLEMIFTPRN
```

*The flexible C-terminal tails were omitted from the models: Residues 503-508 of CYP 17A1 and residues 497-503 of CYP 19A1

Supporting Tables

Table S1: CG simulations performed for CYP 17A1-membrane systems, S1-6, for the full-length wtCYP17 (S1), mtCYP17 (S2, S3), and the globular domain only (S4), and the wild-type (S5) and mutant (S6) TM-helix domain only. CG simulations for CYP 19A1-membrane systems, S7 for wtCYP19 and S8, S9 for mtCYP19, are also listed.

**CG System Identifier**	**System**	**Residues in regions**				**No. of simulations**	**Length of simulations (μs)**
		TM Helix	Linker region	Flexible linker*	Globular domain		
**S1**	wtCYP17	3-19	20-49	20-38	50-502	5	20
**S2**	mtCYP17	3-19	20-49	20-38	50-502	5	20
**S3**	mtCYP17	3-19	20-49	20-49	50-502	5	12-16
**S4**	CYP 17A1 globular domain only				50-502	3	8-10
**S5**	wtCYP17** (residues 1-22)	3-19				10	10
**S6**	mtCYP17**(residues 1-22)	3-19				10	10
**S7**	wtCYP19	18-38	39-54	39-54	55-496	5	8-10
**S8**	mtCYP19		36-54	36-54	55-496	5	8-10
**S9**	mtCYP19		36-54		55-496	5	8-10

*Part of the linker that was not subjected to elastic network restraints in the CG simulations.

**Residues 1-22 were subjected to elastic network restraints.

Table S2. AAMD production simulations performed for CYP 17A1-membrane, CYP 19A1-membrane and CYP 3A4-membrane systems.

AA-MD Simulations	System	CG Simulation	TM-helix	Time (ns)
wtCYP17-AA-IN:1	wtCYP17	wt-CG-S1:4§	in	160
wtCYP17-AA-IN:2	wtCYP17	wt-CG-S1:4§	in	144
mtCYP17-AA-OUT:1	mtCYP17	mt-CG-S2:1*	out	160
mtCYP17-AA-IN:1	mtCYP17	mt-CG-S3:3#	in	148
mtCYP17-AA-IN:2	mtCYP17	mt-CG-S3:3#	in	148
wtCYP19-AA	wtCYP19	S7	in	60
mtCYP19-AA	mtCYP19	S9	-	50
CYP3A4-AA	CYP 3A4	S10	in	90

Note:

§ The starting frame for AAMD was selected from trajectory 4 of system wt-CG-S1 and is the representative frame for all trajectories (wt-CG-S1:1-5). The starting frame was the same for wtCYP17-AA-IN:1 and wtCYP17-AA-IN:2 but different initial velocities were assigned in the two simulations.

*Starting frame for AAMD was taken from the last-frame of trajectory 1 run for system mt-CG-S2

#Starting frame for AAMD was selected from trajectory 3 of system mt-CG-S3 and is the representative frame for trajectories (mt-CG-S3:1-3,5). The starting frame was the same for mtCYP17-AA-IN:1 and mtCYP17-AA-IN:2 but different initial velocities were assigned in the two simulations.

Table S3: Angles and distances characterizing the positioning of the protein in the phospholipid bilayer during CG simulations. Mean and standard deviation values of each parameter are given for full-length mtCYP17, wtCYP17, the globular domain of CYP17, wtCYP19, mtCYP19, and CYP3A4.

CG system	CG simulations	System	Angles (°)		Distances (Å)				TM-helix position
			α	β	TM-helix	Linker	F-G loop	Globular domain	
**S1**	1-5	wtCYP17	101±7	125±7	13±7	20±2	27±4	46±2	in
**S2**	3,5	mtCYP17	104±8	125±11	14±9	22±2	29±4	47±3	in
**S2**	1,2,4	mtCYP17	112±8	122±11	71±9	25±3	34±6	49±3	out
**S3**	1-3,5	mtCYP17	108±9	134±6	15±8	22±2.1	28±2	46±2	in
**S3**	4	mtCYP17	115±7	120±8	85±6	26±2	36±2	50±2	out
**S4**	1-3	CYP17A1 globular domain	105±5	123±6	--	--	27±2	46±2	--
**S5**	1-10	wtCYP17 (residues 1-22)			12±6				in
**S6**	1-10	mtCYP17 (residues 1-22)			--				out
**S7**	1-5	wtCYP19	104 ±5	140 ±6	16 ±8	22 ±2	23 ±2	42 ±2	in
**S8**	1-5	mtCYP19	104±5	152±5	--	24±2	26±2	45±1	no*
**S9**	1-5	mtCYP19	107 ±6	150 ±5	--	35 ±5	26 ±2	44 ±1	no#
**S10**	1-5	CYP3A4	65 ±7	139 ±7	31 ±7	25±4	25±4	42 ±2	in

Note: in=TM-helix embedded in the lipid bilayer core, out= TM-helix gradually drifted out of the bilayer core.
For mtCYP19, no* : No TM-helix was present and the linker region was lying on the membrane (not similar to crystal structure); no# : No TM-helix was present and the position of the linker region was similar to the crystal structure (PDB: 4KQ8).
**For mtCYP17 and wtCYP17, the residue ranges for the angle and distance computations were: TM-helix: 3-19, linker: 20-49, F-G loop: 210-227, globular domain: 50-502. For wtCYP19, the ranges were: TM-helix: 18-38, linker: 39-54, F-G loop: 230-237, globular domain: 55-496. For mtCYP19, the linker was: 36-54.

Supporting Figures

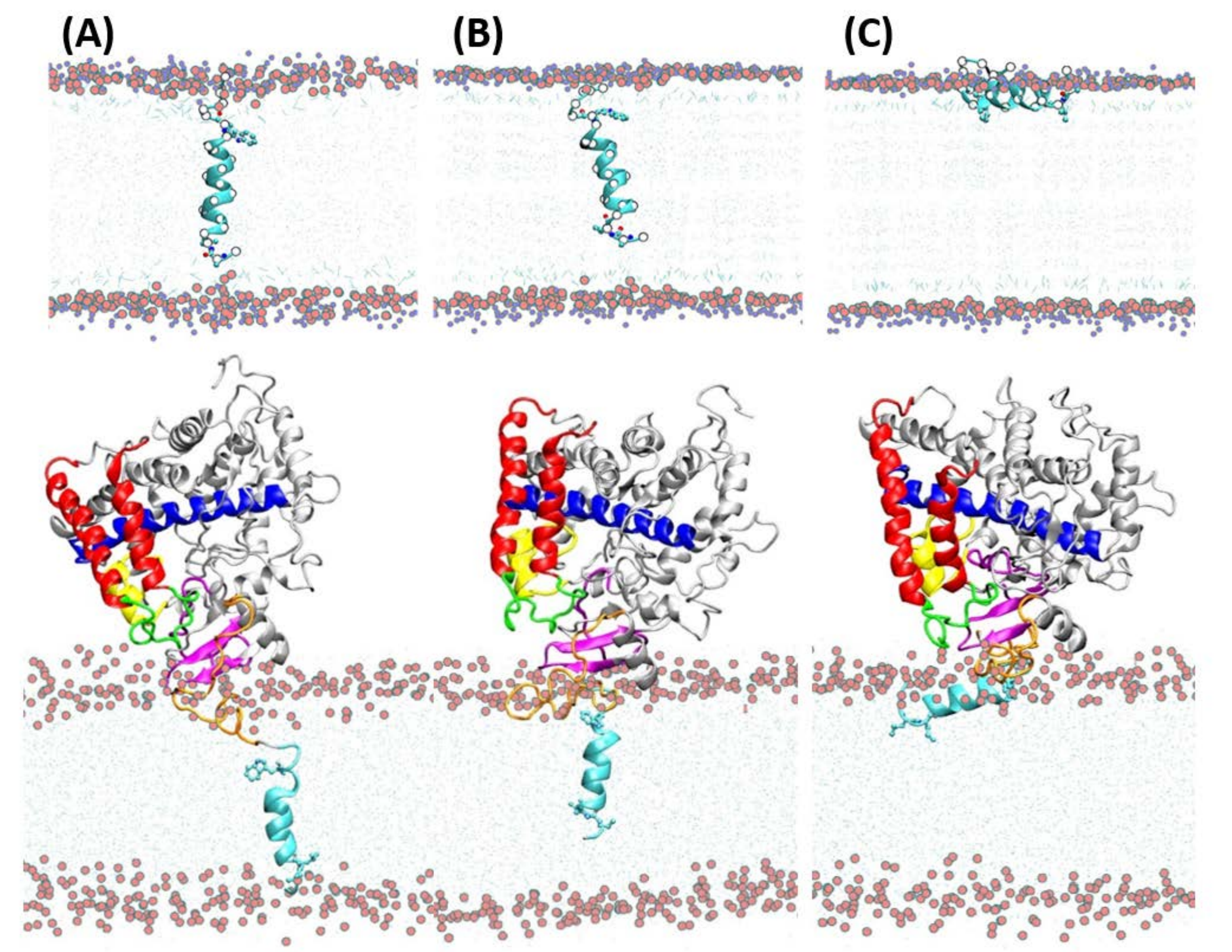

Figure S1: The gradual drifting of the mtCYP17 TM-helix out of the membrane core. The mutant TM-helix forms unstable interactions in simulations of both TM-helix only (snapshots from trajectory mt-CG-S6:2 at 2, 591 and 1208 ns, top left to right), where the complete mtCYP17 TM-helix went to the headgroup region, and full-length mtCYP17 (snapshots from trajectory mt-CG-S2:1 at 0, 270 and 8100 ns, bottom left to right). The TM-helix is shown in cyan, the I-helix in blue, the F and G helices in red, the F-G loop in green, and the B-C loop in yellow. The flexible linker between the TM-helix and the globular domain is shown in orange. The red spheres represent phosphate atoms in the headgroups of the POPC lipid bilayer.

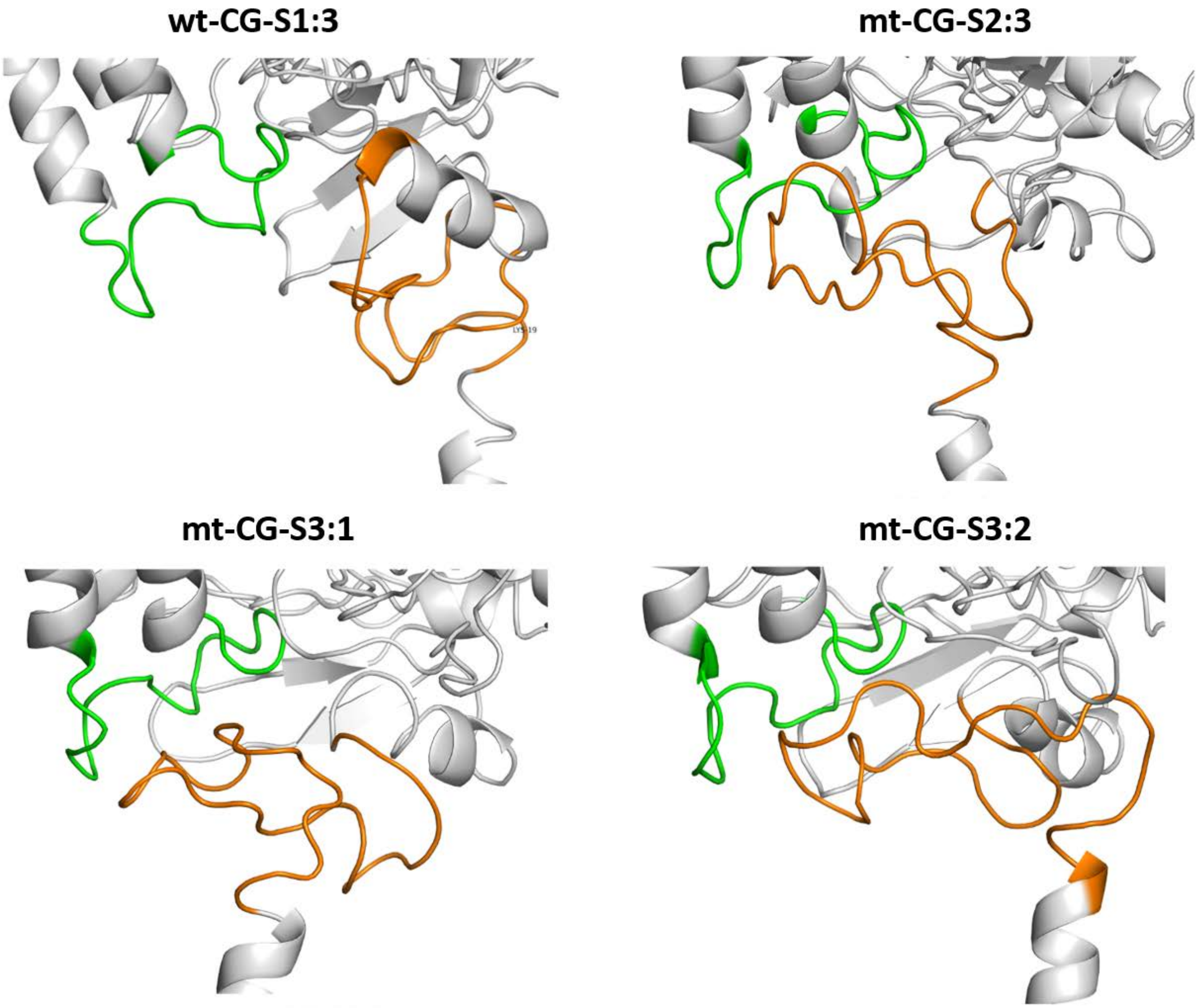

Figure S2: Linker position after back-conversion of wtCYP17 and mtCYP17 CG frames to an all-atom representation. The linker (orange) position is compared for wtCYP17 (wt-CG-S1:3) and mtCYP17 (mt-CG-S2:3, mt-CG-S3:1,2) with respect to the F-G loop (green). In mtCYP17, when the TM-helix remained inside the membrane core, the linker was seen to form strong polar contacts with the F-G region. When the linker position was similar to that in wtCYP17 (wt-CG-S1:3), the TM-helix drifted out of the membrane core (mt-CG-S2:1).

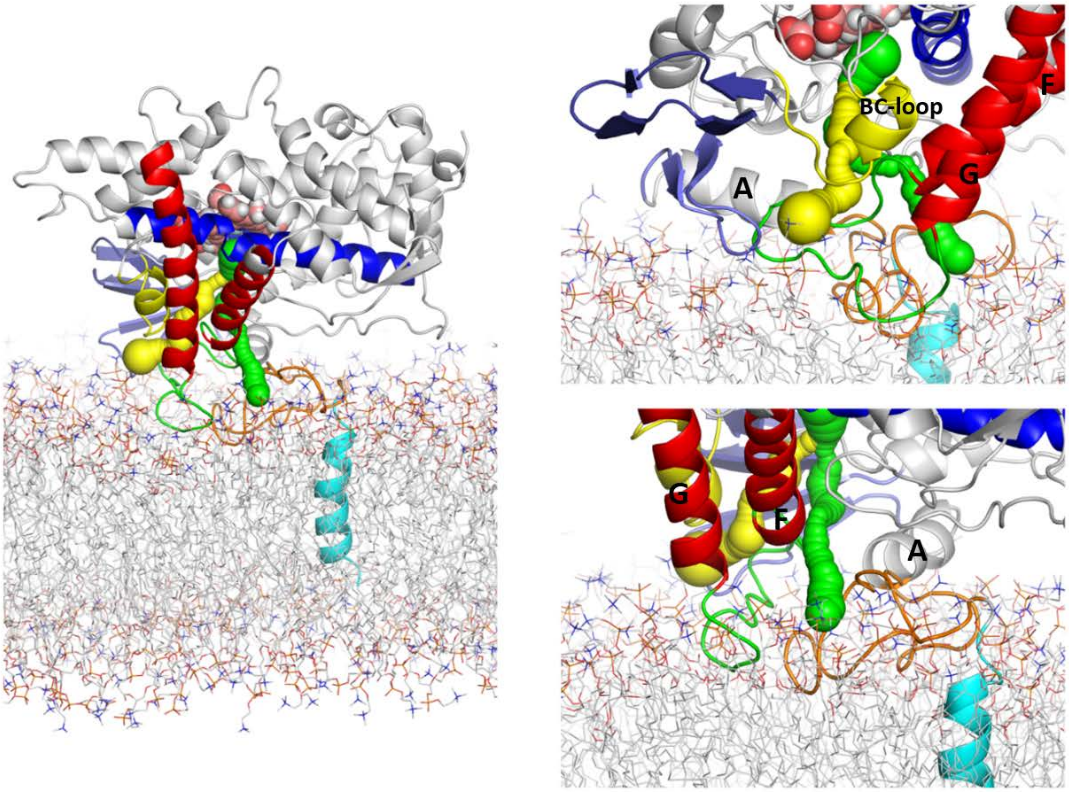

Figure S3: mtCYP17 shown with ligand access tunnels computed from AA-MD trajectories. Ligand access tunnels 2b (yellow spheres), 2d and 2f (green spheres) are shown with respect to the linker position (protein colored as in Figure 1). Close-ups in two orientations rotated by about 90 degrees around the z-axis are shown on the right. The trapped linker (orange) could potentially hinder the ligand access tunnels from opening through the membrane into the protein's binding pocket.

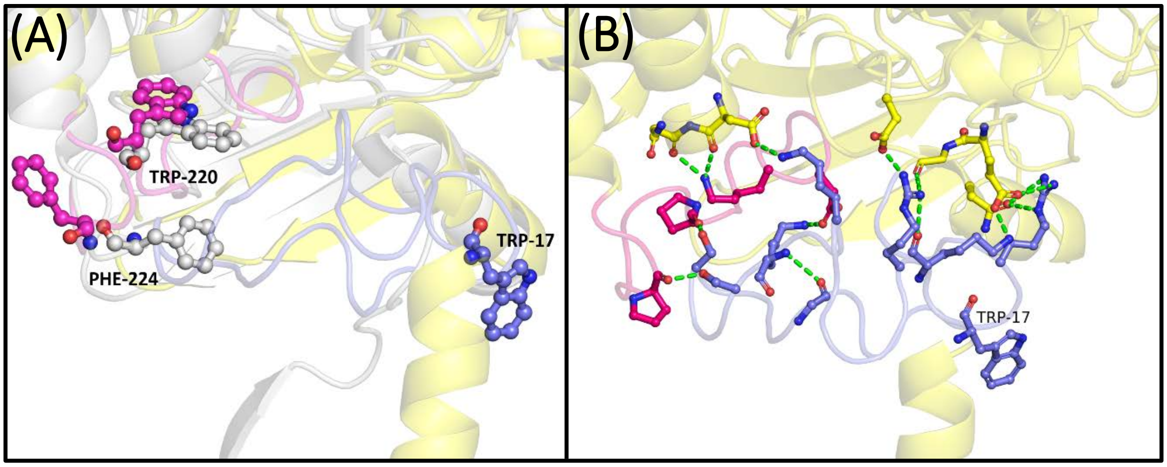

Figure S4: Initial representative frame after back-conversion of mtCYP17 (mt-CG-S3:3) (yellow) superimposed on X-ray crystal structure in grey (PDB 3RUK) to highlight differences in the linker and F-G loop conformations. (A, left) The difference in the F-G loop orientation shown in pink for mtCYP17 after back-conversion and grey for X-ray crystal structure and the changes in the side chain conformations of F224 and W220 are shown in stick representation. (B, right) The polar interactions in the mtCYP17 structure between the linker (blue), A helix, F helix (yellow), F-G loop region (pink) and beta-sheet (residue 480-485) are shown by green dashed lines. The side chains are colored by atom type.

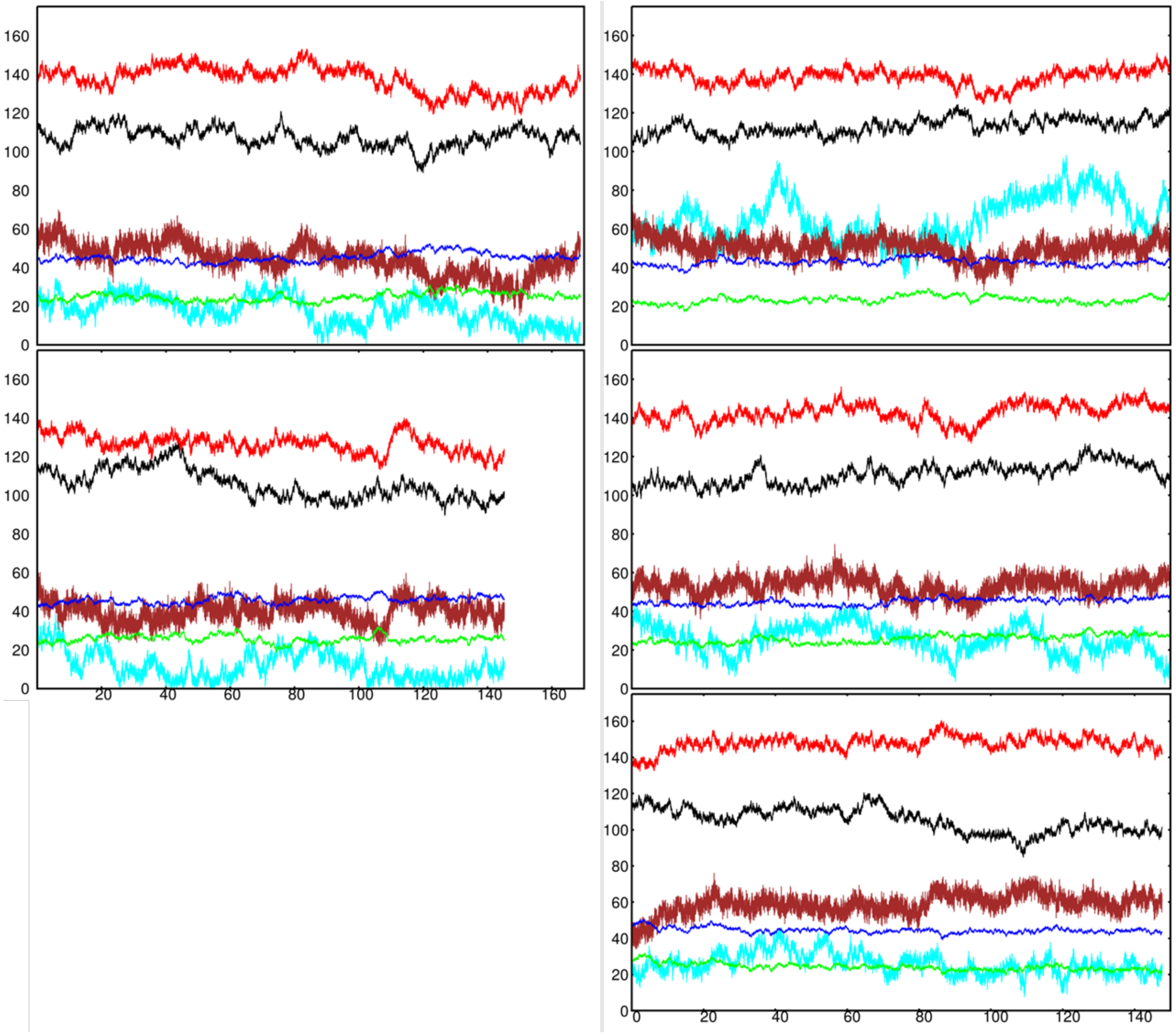

Figure S5: Evolution of the position of CYP 17A1 with respect to the phospholipid bilayer during AAMD. Plots of distances and angles (y-axis) against time (x-axis), characterizing the position and orientation are given (from top to bottom) for trajectories wtCYP17-AA-IN:1-2 (left), and mtCYP17-AA-OUT:1 and mtCYP17-AA-IN:1-2 (right). Angles: α (black), β (red), heme-tilt (brown), TM-helix tilt (cyan). Distances: globular domain center-of-mass (CoM) (blue) and F-G loop CoM (green) to the membrane CoM are shown.

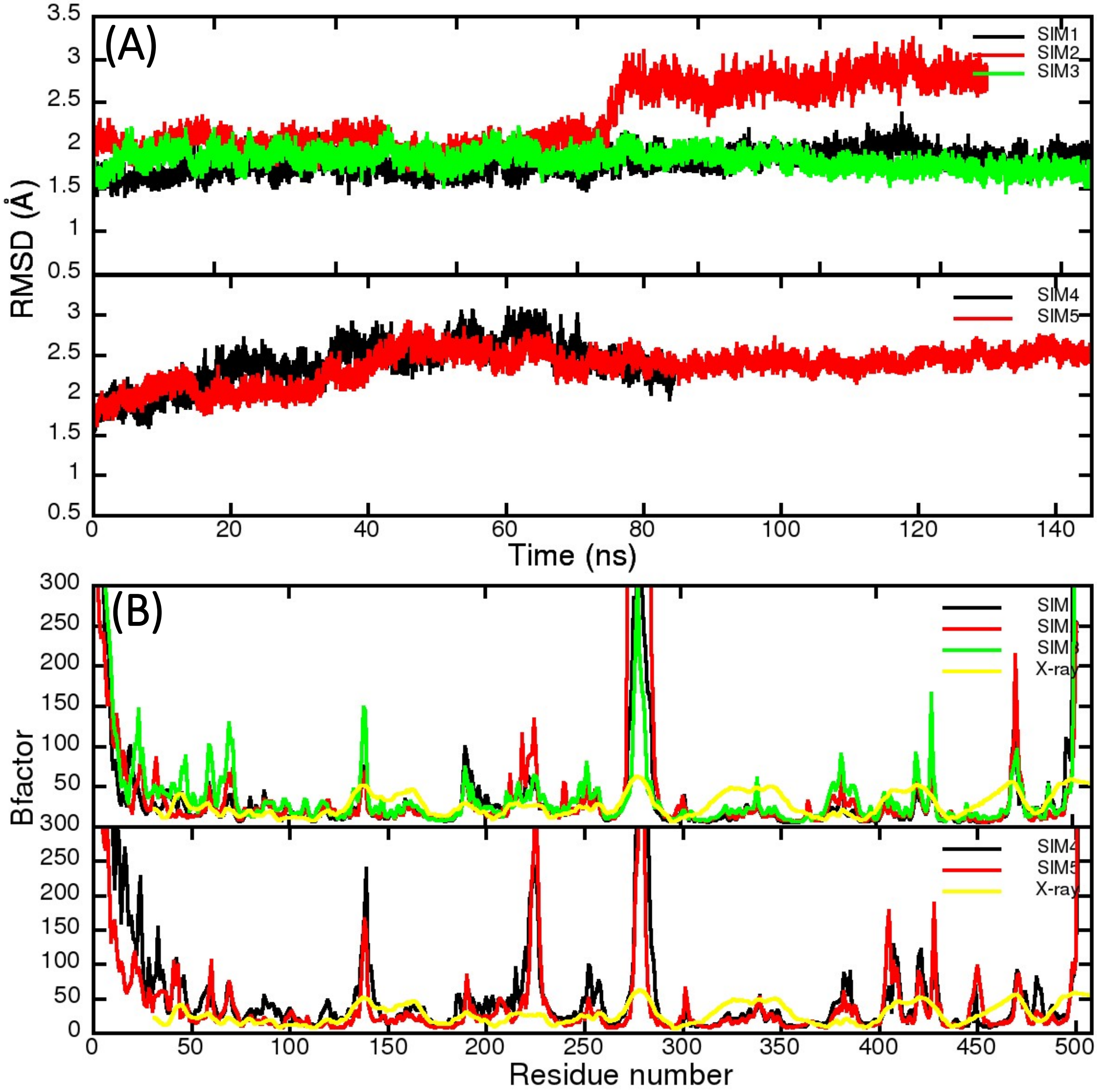

Figure S6: Cα atom RMSD and B-factor values in AAMD simulations of CYP 17A1. (A) RMSDs with respect to the minimized crystal structure are shown along the simulations for mtCYP17 (for mtCYP17-AA-OUT:1 (black), mtCYP17-AA-IN:1 (red) and 3 (green)), (upper plot) and for wtCYP17 (wtCYP17-AA-IN:1 (black) and 5 (red)) (lower plot). (B) The average B-factor values (mean squared atomic positional fluctuations multiplied by $8\pi^3/3$ Å^2) along the protein sequence are compared with crystallographic B-factor values (yellow). In the mtCYP17-AA-IN:1 simulation, the RMSD increased quite abruptly from 2 Å to 3 Å after ~80 ns, and then remained stable. This increase in RMSD was due to the H-I (residues 276-288) loop being highly flexible, as shown by the computed and experimental B-factors.
